# Traditional Chinese Medicine for Bradyarrhythmia: Evidence and Potential Mechanisms

**DOI:** 10.3389/fphar.2018.00324

**Published:** 2018-04-09

**Authors:** Shuo Liu, Guihua Tian, Jing Chen, Xiaoyu Zhang, Aiming Wu, Min Li, Yang Sun, Baoshan Liu, Yanwei Xing, Hongcai Shang

**Affiliations:** ^1^Key Laboratory of Chinese Internal Medicine of Ministry of Education and Beijing, Dongzhimen Hospital Affiliated to Beijing University of Chinese Medicine, Beijing, China; ^2^Chinese Cochrane Center, West China Hospital, Sichuan University, Chengdu, China; ^3^Baokang Hospital Affiliated to Tianjin University of Traditional Chinese Medicine, Tianjin, China; ^4^Tianjin Medical University General Hospital, Tianjin, China; ^5^Guang'anmen Hospital, Chinese Academy of Chinese Medical Sciences, Beijing, China

**Keywords:** Traditional Chinese Medicine, Bradyarrhythmia, evidence-based medicine, mechanisms, systematic reviews and meta-analyses

## Abstract

**Importance:** The incidence of Bradyarrhythmias is high among the population. However, at early stages of the disease, it cannot always get enough attention and is lack of safe and effective therapies, until it is serious enough to resort to pacemaker implantation. Traditional Chinese Medicine (TCM) has a long history of treating Bradyarrhythmia, with a lot of formulas being widely used in clinical practice. While the effectiveness and the underlying mechanisms of these formulas have not yet been clearly identified.

**Objective:** To evaluate the effectiveness of some common TCM formulas in treating patients with Bradyarrhythmia and to summarize the current evidence as to their mechanisms.

**Data Sources:** Relevant studies were identified by searching for papers published from January 2000 to August 2017 in Pubmed; EMBASE; the Cochrane Library (Cochrane Central Register of Controlled Trials); the China National Knowledge Internet; and the China biology medicine, Wanfang, and VIP databases. The following medical subject heading (MeSH) terms were included for Pubmed search and adapted for other databases as needed-“Medicine, Chinese Traditional,” “Bradycardia.”

**Study Selection:** Randomized clinical trials investigating treatment outcomes in Bradyarrhythmia patients with one of the six TCM formulas (Shenxian-shengmai oral liquid, Shensong Yangxin capsule, XinBao pill, Mahuang-Fuzi-Xixin decoction, Zhigancao decoction and Shengmai injection).

**Data Extraction and Synthesis:** Two independent reviewers performed the data extraction and assessed study quality. A meta-analysis was performed to calculate risk ratio (RR) and 95% confidence index (CI) using random-effects and fixed-effects model.

**Results:** A total of 121 clinical trials with 11138 patients were included. Of the six TCM formulas, SXSM (RR:1.33, 95% CI 1.27 to 1.39, *P* < 0.00001), SSYX (RR:1.52, 95% CI 1.40 to 1.66, *P* < 0.00001), XB can be more effective than common treatment (RR 1.18, 95% CI 1.11 to 1.26, *P* < 0.00001), as well as placebo (RR 5.33, 95% CI 2.88-9.87, *P* < 0.00001), but less effective than TCM dialectical therapy (RR:0.75, 95% CI 0.68 to 0.82, *P* < 0.00001). Compared to the control group, MFX (RR:1.30, 95%CI 1.23 to 1.37, *P* < 0.00001), ZGC (RR:1.35, 95%CI 1.23 to 1.48, *P* < 0.00001), SMI (RR:1.36, 95%CI 1.21 to 1.52, *P* < 0.00001) can be more effective. The overall quality of the included trials were relatively low, with the limitations of small sample size, inadequate descriptions in randomization, allocation concealment and blinding methods.

**Conclusions and Relevance:** There are evidence that some TCM formulas might help to relieve Bradyarrhythmias. But with the relatively low quality of the clinical trials and mechanism studies, we still need more high-quality researches to verify the conclusions.

## Introduction

Bradyarrhythmias is a common arrhythmia encountered in clinical practice. Fatigability, reduced exercise capacity and symptoms of heart failure (HF) are most familiar signs of the persistent Bradyarrhythmia, along with some subtle symptoms such as irritability, lassitude, inability to concentrate, apathy, forgetfulness and dizziness. Dizziness, pre-syncope and syncope are common symptoms with intermittent severe forms of Bradyarrhythmias and are due to a sudden decrease in cerebral blood flow (Brignole et al., 2013). According to the survey from Tresch, in the Baltimore Longitudinal Study of Aging, the prevalence of unexplained Sinus bradycardia was approximately 4% and was nearly identical in men and women (Tresch and Fleg, 1986). With the development of technology, pacemaker implantation is widely used in treating different kinds of Bradyarrhythmias. In Dublin, the pacemaker implantation rate was 0.6% (Keaney et al., 2013). However, with the constraints of unbalanced economy and technology, the use of pacemaker implantation still cannot sufficiently meet the clinical needs (Baman et al., 2010). As for the patients with abnormal low heart rate, accompanied with symptoms of palpitation, panting and fainting, or patients with pacemaker contraindications, there are no safe and effective treatment from modern medicine so far.

TCM has a long history of treating Bradyarrhythmia, which may start from as early as the Han Dynasty in China (about 2,000 years ago). A Chinese physician named Zhang Zhongjing first gave the therapies for the symptoms the same as those of Bradyarrhythmia, which was recorded in the TCM classics *Treatise on Febrile and Miscellaneous Diseases (Shang Han Lun in Chinese)*. Actually, there was no specific term for Bradyarrhythmia at that time, the clinicians assigned it as palpitation or slow pulse. For the pathogenesis, TCM physicians take it as stasis of blood because of Qi or Yang deficiency. Accordingly, TCM formulas are aiming to reinforcing Qi and warming Yang. Based on modern pharmacological research, these formulas can be effective alleviating Bradyarrhythmia by regulating sympathetic and parasympathetic nervous system, restraining myocardial collagen hyperplasia and fibrosis, reducing inflammation and increasing antioxidant activity, regulating myocardial energy metabolism and ion channels.

Nowadays, TCM is playing an important role in treating Bradyarrhythmia. For ease of use, TCM decoction has been developed into a variety of dosage forms, such as capsule, dropping pill, oral liquid and injection. What's more, TCM is increasingly welcomed in many developed countries, such as Australia and the United States (Hao et al., 2017). Therefore, to evaluate the treatment effect and identify potential mechanisms of TCM formulas for Bradyarrhythmias, we searched six of the most often used formulas (SXSM, SSYX, XB, MFX, ZGC, SMI) and gave this systematic review.

## The search for and selection of RCTs

### Data sources and searches

Relevant studies were identified by searching for papers published from January 2000 to August 2017 in Pubmed; EMBASE; the Cochrane Library (Cochrane Central Register of Controlled Trials); the China National Knowledge Internet; and the China biology medicine, Wanfang, and VIP databases. The following medical subject heading (MeSH) terms were included for Pubmed search and adapted for other databases as needed- “Medicine, Chinese Traditional”, “Bradycardia”; The search algorithm for MEDLINE was as follows: (((((((Traditional Chinese Medicine[Title/Abstract]) OR Chinese proprietary medicine[Title/Abstract])) OR “Medicine, Chinese Traditional”[Mesh])) OR ((((((((shenxian shengmai[Title/Abstract]) OR ((mahuang fuzi xixin[Title/Abstract]) OR zhigancao [Title/Abstract])) OR xin bao pill[Title/Abstract]) OR shensongyangxin[Title/Abstract]))) AND ((((((((((((Bradycardia[Title/Abstract]) OR Brugada Syndrome[Title/Abstract]) OR Heart Block[Title/Abstract]) OR Long QT Syndrome[Title/Abstract]))). Similar but adapted search terms were used for other databases of published reports or search engines. The reference lists of all retrieved papers were checked for other potentially relevant citations, and studies not included in the electronic sources mentioned previously were searched manually.

### Study selection

We included reports of clinical studies with the following criteria: (1) Randomized clinical trials treating Bradyarrhythmia using TCM formulas (SXSM, SSYX, XB, MFX, ZGC, SMI), with no language limitation; (2) Studies reporting efficacy outcomes (healed, markedly effective, effective and ineffective). We excluded reports of studies with the following features: (1) Two or more TCM formulas vs. conventional therapies; (2) Studies with flaws such as inappropriate design, incomplete or wrong results; (3) TCM formulas vs. cardiac pacemaker; (4) Conference paper and academic dissertation; (5) Studies with patients less than 20 per group.

### Data extraction and quality assessment

Two authors (SL, JC) reviewed the trials to ensure that they met inclusion criteria, abstracted the data and this was checked for accuracy by the other authors. Disagreements were resolved by consensus-based discussion. We performed objective assessment of the trials using the methods specified in the Cochrane Handbook of Systematic Reviews assessing for risks of bias (selection bias, performance bias, detection bias, attrition bias, reporting bias).

**Figure 1:**
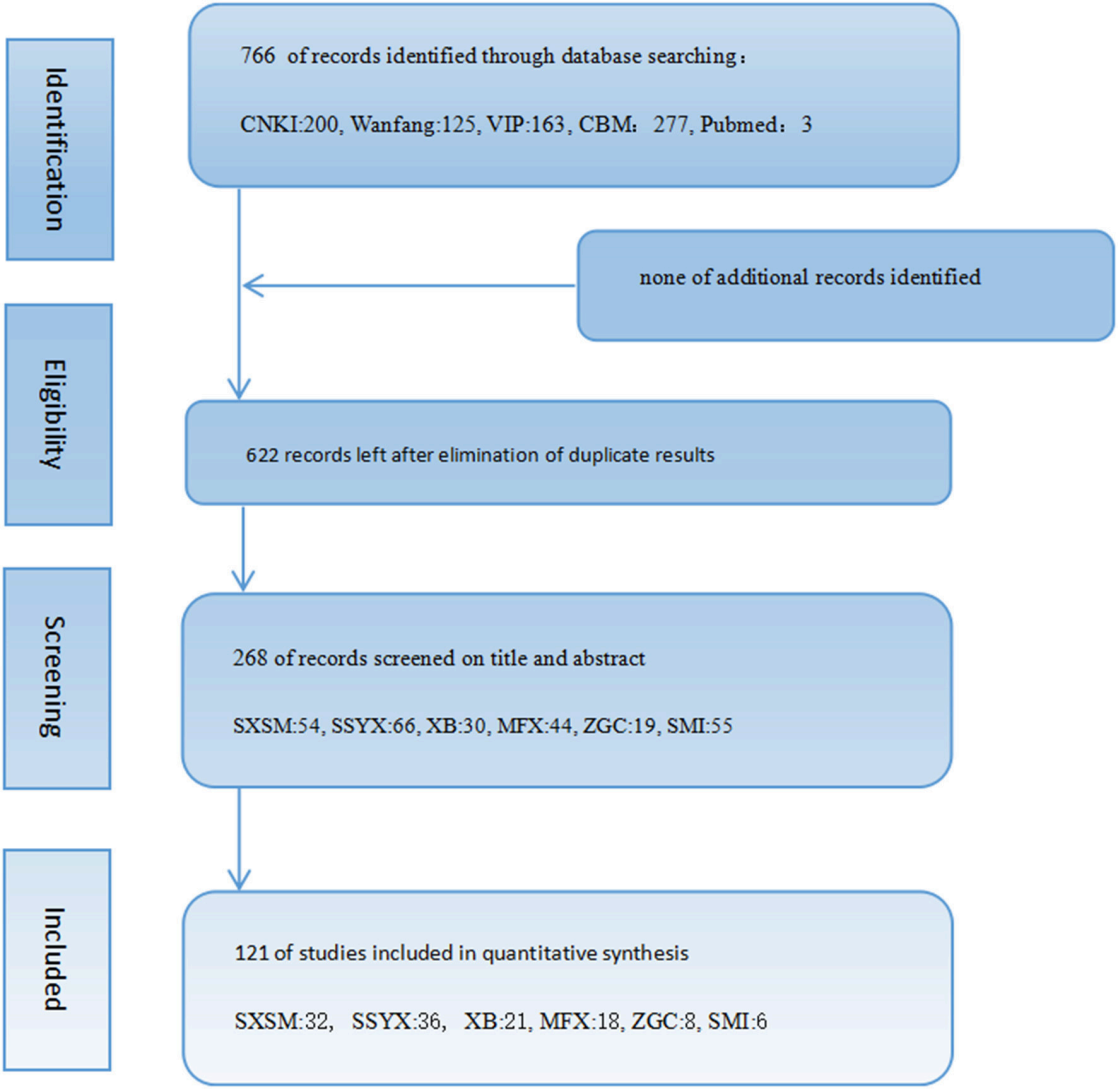
Flowchart of the included studies' selection process.

The initial search yielded 766 records, 763 in Chinese and 3 in English. After elimination of duplicate results, 268 articles with six formulas remained. Finally, 121 articles were reviewed and assessed. There were 32 of SXSM, 36 of SSYX, 21 of XB, 18 of MFX, 8 of ZGC, 6 of SMI (Figure 1).

### Data synthesis and analysis

Meta-analysis was performed according to the recommendations of Cochrane collaboration and in line with the PRISMA statement (Liberati et al., 2009). To assess effective rate, “cured” and “marked effective” cases are both seen as effective cases. All these analyses were performed using the Cochrane RevMan 5.3 program. Pooled treatment effects were estimated using relative risk (RR) with 95% confidence interval (CI), calculated by random-effects and fixed-effects model. Heterogeneity was assessed by chi-square tests and the *I*^2^ statistic-we defined *I*^2^ < 50% to be low heterogeneity, referring to the Cochrane Handbook of Systematic Reviews. Publication bias was estimated using funnel plots.

### Study characteristics

A total of 121 clinical trials with 11,138 patients were included. 2,330 patients were from studies of SXSM, 4660 from SSYX, 1700 from XB, 1398 from MFX, 651 from ZGC, 399 from SMI (Table 1) (details of study characteristics were offered as Supplementary Material).

**Table 1:** Components of formulas and mechanism of effect.

**Name of formula**	**Accepted name**	**Main components**	**Mechanism of effect**	**References**
Shenxian-shengmai oral liquid (SXSM)	Panax ginseng C.A.Mey. Epimedium brevicornu Maxim. Cullen corylifolium (L.) Medik. *Lycium barbarum* L. Ephedra sinica Stapf Asarum sieboldii Miq. Salvia miltiorrhiza Bunge Whitmania pigra Whitman	Ginsenosides, Ephedrine Hydrochloride, Psoralen, psoralen, icariin, protocatechuic aldehyde	Regulate parasympathetic nervous system	Liu et al., 2017
Shensong Yangxin Capsule (SSYX)	Panax ginseng C.A.Mey. Ophiopogon japonicus (Thunb.) Ker Gawl. Cornus officinalis Siebold & Zucc. Salvia miltiorrhiza Bunge Ziziphus jujuba Mill. Taxillus sutchuenensis (Lecomte) Danser Paeonia anomala subsp. veitchii (Lynch) D.Y.Hong & K.Y.Pan Eupolyphagasinensis Walker Nardostachys jatamansi (D.Don) DC. Coptis chinensis Franch. Kadsura longipedunculata Finet & Gagnep.; OsDraconis	saponins, phenolic acids, tanshinones, lignans, terpenoids, alkaloids and flavonoids, according to their chemical structures	restrain Myocardial collagen proliferation and cardiac fibrosis	Shen et al., 2014; Liu et al., 2015; Dang et al., 2016
XinBao pill (XB)	Datura metel L. Cervus nippon Panax ginseng C.A.Mey. Aconitum carmichaeli Debeaux Cinnamomum cassia (L.) J.Presl Panax notoginseng (Burkill) F.H.Chen Abelmoschus moschatus Medik. Venenum Bufonis DryobalanopsaromaticaGwaertn.f.	atropine, scopolamine, Ginsenosides, Total velvet-antler polypeptide, Aconite normal butanol	M2 receptor antagonism	Shen et al., 2014; Liu et al., 2015; Dang et al., 2016
Mahuang-Fuzi-Xixin decoction (MFX)	Ephedra sinica Stapf Aconitum carmichaeli Debeaux Asarum sieboldii Miq.	methyl ephedrine, aconine, songrine, fuziline, neoline, talatisamine, chasmanine, benzoylmesaconine, benzoylaconine and benzoylhypaconine	reducing inflammation and increasing antioxidant activities	Tang et al., 2015; Sun et al., 2016
Zhigancao decoction (ZGC)	Glycyrrhiza uralensis Fisch. ex DC. Zingiber officinale Roscoe Cinnamomum cassia (L.) J.Presl Panax ginseng C.A.Mey. Rehmannia glutinosa (Gaertn.) DC. Asini Corii Colla Ophiopogon japonicus (Thunb.) Ker Gawl. Cannabis sativa L. Ziziphus jujuba Mill.	uncertain	It may be related to the content of Ca^2+^ in muscle tissue or excitability of M receptor	Hai et al., 2017
Shengmai injection (SMI)	Panax ginseng C.A.Mey. Ophiopogon japonicus (Thunb.) Ker Gawl. Schisandra chinensis (Turcz.) Baill.	ginsenosides, lignans, steroidal saponins and homoiso-flavanones	modulate the myocardial energy metabolism	Wu et al., 2011; Zhan et al., 2015

**Figure 2:**
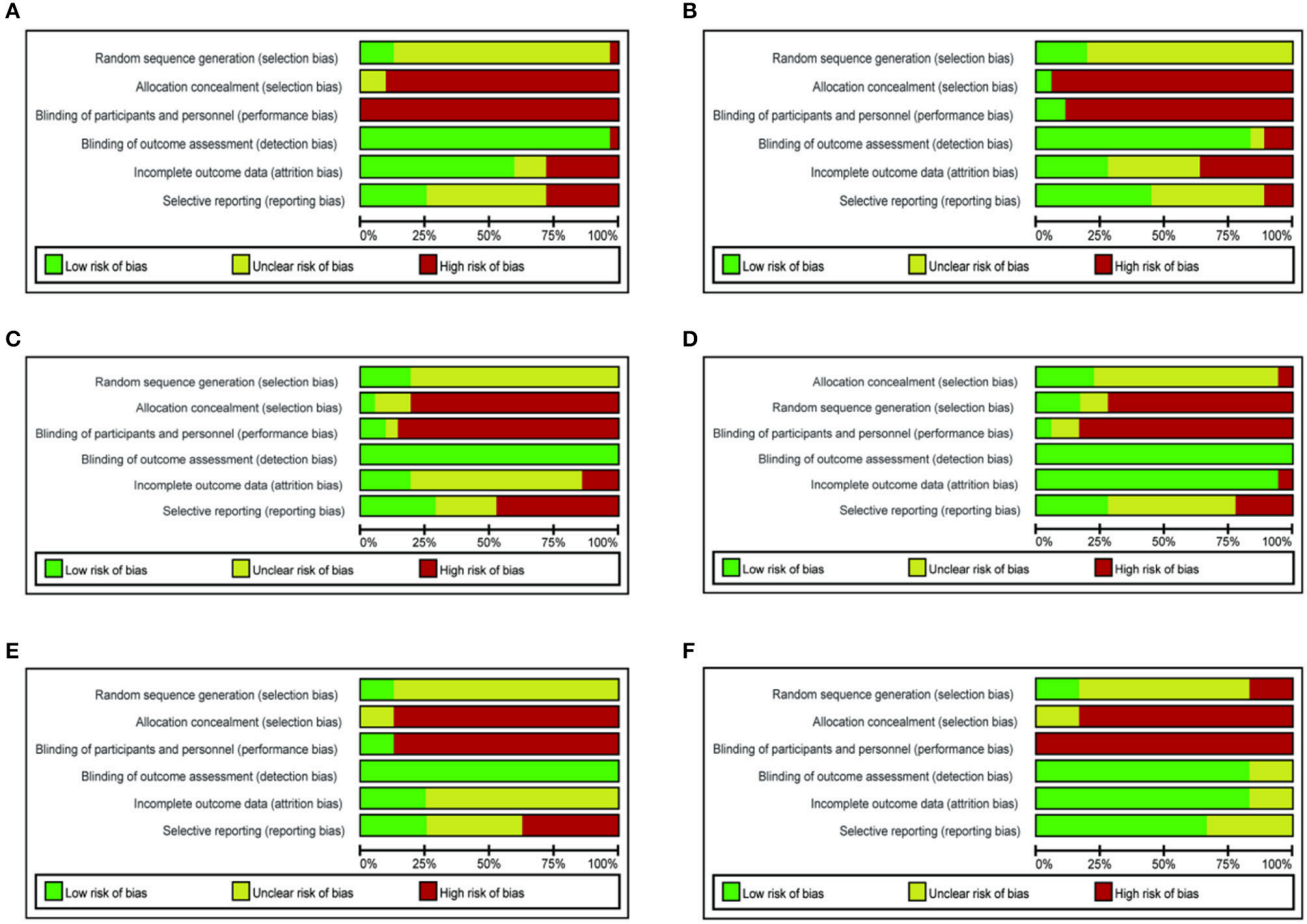
Risk of bias graph **(A)** SXSM, **(B)** SSYX, **(C)** XB, **(D)** MFX, **(E)** ZGC, **(F)** SMI.

Most of the studies only referred randomized allocation without specific randomization method. Only a few studies mentioned blinding of participants or outcome assessors. Most studies provided data of diagnostic standards and evaluation criterion (Figure 2). The time of publication of these 121 studies ranged from 2004 to 2017, 74 of which introduced the occurrence of side effects.

## TCM for bradyarrhythmia evidence and mechanisms

### Shenxian-shengmai oral liquid

SXSM is prepared from 8 herbs, namely red ginseng, epimedium, psoralen, medlar, ephedra, asarum, salvia, and leech (Table 1). It has been listed in the Chinese national directory of health insurance in 2017. Animal experiments showed that SXSM can increase heart rate by inhibiting heart parasympathetic transmission based on the decreased CHRNA2 (encodes nicotinic acetylcholine receptor) and increased ACE-1 (encodes acetylcholinesterase). SXSM upregulated ATP2A1 and FKBP1B, therefore, restored Ca^2+^ stores induced by restored expression of SERCA2a and FKBP12.6 contributed directly to increased heart rate. In addition, in ventricular myocardium, SXSM increased the supply of ATP by enhancing TCA cycle and oxidation-respiratory chain. It also upregulated proteins ranged from enzymes of TCA cycle to subunits of complex I and ATP synthase (Liu et al., 2017).

**Figure 3:**
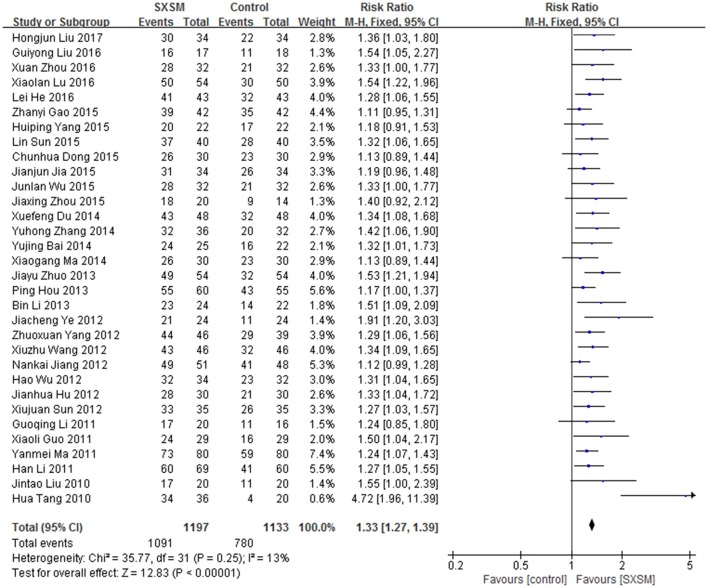
SXSM for Bradyarrhythmia efficacy by meta-analysis.

Studies (Liu and Li, 2010; Tang et al., 2010; Guo, 2011; Li G, 2011; Li H, 2011; Ma, 2011; Hu J. et al., 2012; Jiang et al., 2012; Sun et al., 2012; Wang and Liu, 2012; Wu et al., 2012, 2015; Yang et al., 2012; Ye, 2012; Hou et al., 2013; Li B, 2013; Zhuo, 2013; Bai and Hou, 2014; Du, 2014; Ma and Dong, 2014; Zhang, 2014; Dong and Ma, 2015; Gao et al., 2015; Jia, 2015; Sun and Luo, 2015; Yang and Ren, 2015; Zhou, 2015; He, 2016; Liu et al., 2016; Lu et al., 2016; Zhou et al., 2016; Liu H, 2017) of SXSM were included. Courses of the treatment ranged from 1 to 8 weeks. There were 2330 patients involved, including 1,197 in SXSM group (1,091 effective cases) and 1,133 in control group (780 effective cases). Meta-analyse was performed with a fixed-effect model as no significant heterogeneity was found (*I*^2^ < 50%, *P* > 0.1). It showed that SXSM was effective in treating Bradyarrhythmia (RR: 1.33, 95% CI 1.27 to 1.39, *P* < 0.00001) (Figure 3).

### Shensong yangxin capsule

SSYX is prepared from 12 ingredients such as Panax ginseng, dwarf lilyturf tuber, nardostachys root, etc. (Table 1). It was listed in the Chinese national directory of health insurance in 2009. Mass spectrometric and chromatographic detection identified major constituents including the saponins, phenolic acids, tanshinones, lignans, terpenoids, alkaloids, and flavonoids, according to their chemical structures (Liu et al., 2015). Animal experiments showed thatmRNA levels of TGF-β1, col-1, col-3, MMP-2, MMP-9 and α-SMA were downregulated, whereas Smad7 expression was upregulated after treatment with SSYX in rats with cardiac fibrosis (Shen et al., 2014). Meanwhile, SSYX can downregulated the level of ColI and ColIII, restrain Myocardial collagen proliferation (Dang et al., 2016). After blockage of the autonomic nervous system with metoprolol and atropin, SSYX had no effect on intrinsic HR (IHR), but decreased corrected sinus node recovery time (CSNRT) and sinus atrium conducting time (SACT). In isolated guinea pig ventricular myocytes, the most obvious effect of SSYX on action potential was a shortening of the action potential duration (APD) without change in shape of action potential.

**Figure 4:**
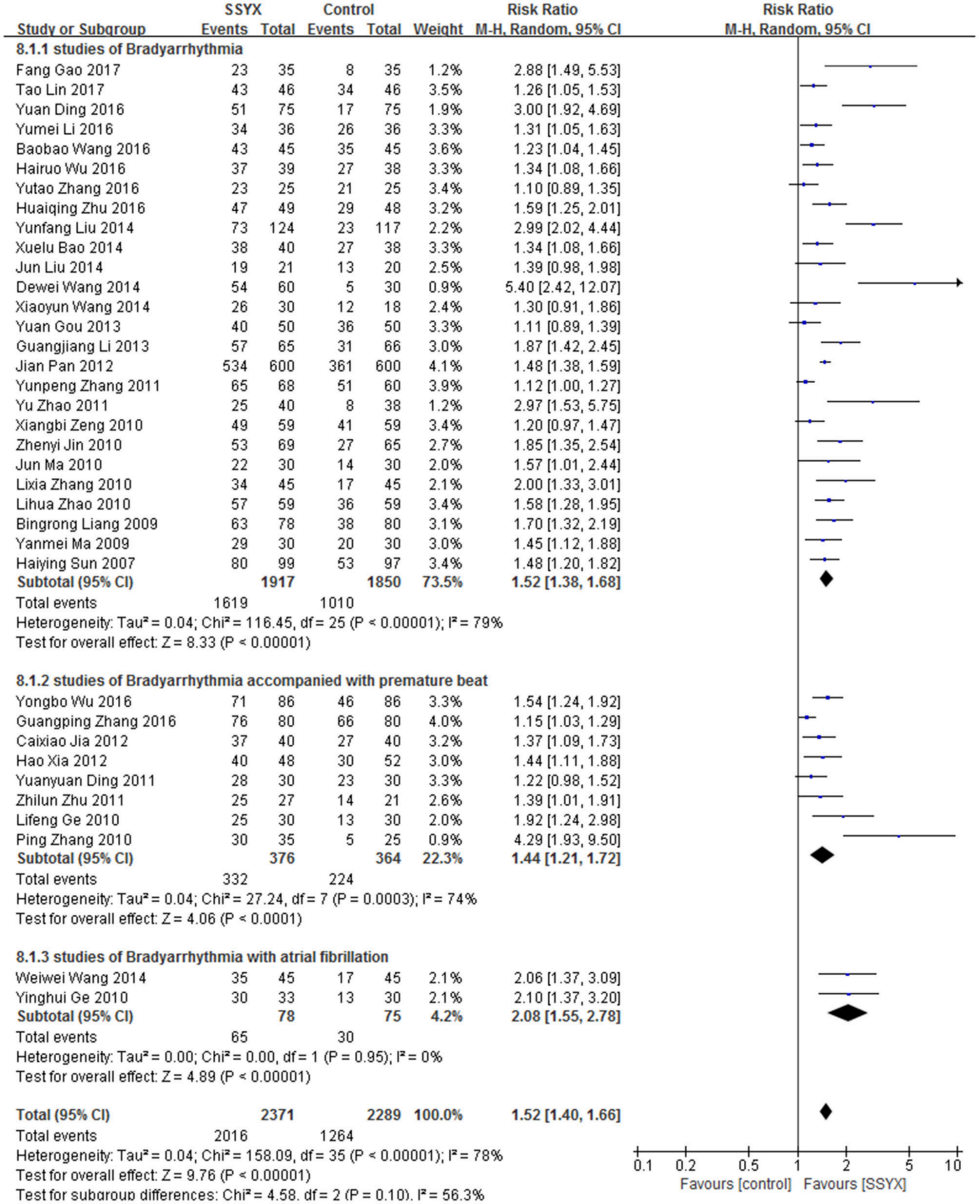
SSYX for Bradyarrhythmia efficacy by meta-analysis.

Thirty-six studies (Sun and Liu, 2007; Liang, 2009; Ma, 2009; Ge L., 2010; Ge Y., 2010; Jin et al., 2010; Ma and Zhu, 2010; Zeng et al., 2010; Zhang, 2010, 2016,a,b; Zhang et al., 2010, 2011; Zhao, 2010; Ding et al., 2011; Zhao et al., 2011; Zhu, 2011, 2016; Jia and Wang, 2012; Pan and Cui, 2012; Xia et al., 2012; Gou, 2013; Li, 2013; Bao and Li, 2014; Liu et al., 2014,a,b; Wang D. et al., 2014; Wang W. et al., 2014; Wang X., 2014; Ding, 2016; Li et al., 2016; Wang, 2016; Wu, 2016; Wu et al., 2016; Gao, 2017; Lin, 2017) of SSYX were included. Courses of the treatment ranged from 2 weeks to 6 months. There were 4,660 patients involved, including 2,371 in SSYX group (2,016 effective cases) and 2,289 in control group (1,264 effective cases). Meta-analyse was performed with a randomized-effect model as heterogeneity was found (*I*^2^ > 50%, *P* < 0.10). Pooled result demonstrated that SSYX treatment is more effective than control treatment (RR:1.52, 95% CI 1.40 to 1.66, *P* < 0.00001). Subgroup analysis was made according to the types of Bradyarrhythmia. There were 26 studies of Bradyarrhythmia, 8 studies of Bradyarrhythmia accompanied with premature beat, 2 studies of Bradyarrhythmia with atrial fibrillation. Subgroup analysis demonstrated that differences between the 3 types of Bradyarrhythmia were not obvious (*I*^2^ = 56.3%, *P* = 0.10). And SSYX is effective in treating any of the 3 types of Bradyarrhythmia (*P* < 0.05) (Figure 4).

### Xinbao pill

XB consists of flos daturae, cornu cervi, ginseng, radix aconiti carmichaeli, etc. (Table 1). There were few basic research on this formula as a whole. The main active ingredient of flos daturae is atropine, whichis commonly used as an emergency medicine for improving heart rate (Gao et al., 2005). Cornu cervi extract canactivate the pi3k-akt signaling pathway which has important effects on the function of the heart (Zálesák et al., 2015; Zhang et al., 2016). As one of the main components of ginseng, Ginsenoside Rg5 promotes Angiogenesis and Vasorelaxation by Specific Activation of Insulin-like Growth Factor-1 Receptor. These findings revealed a mechanism for the positive regulation of vascular function (Cho et al., 2015). What's more, it was noted that Ginsenoside Rg2 can alleviate nervous system side effects caused by atropine in flos daturae, which may imply the advantage of TCM compatibility (Yang et al., 2009).

**Figure 5:**
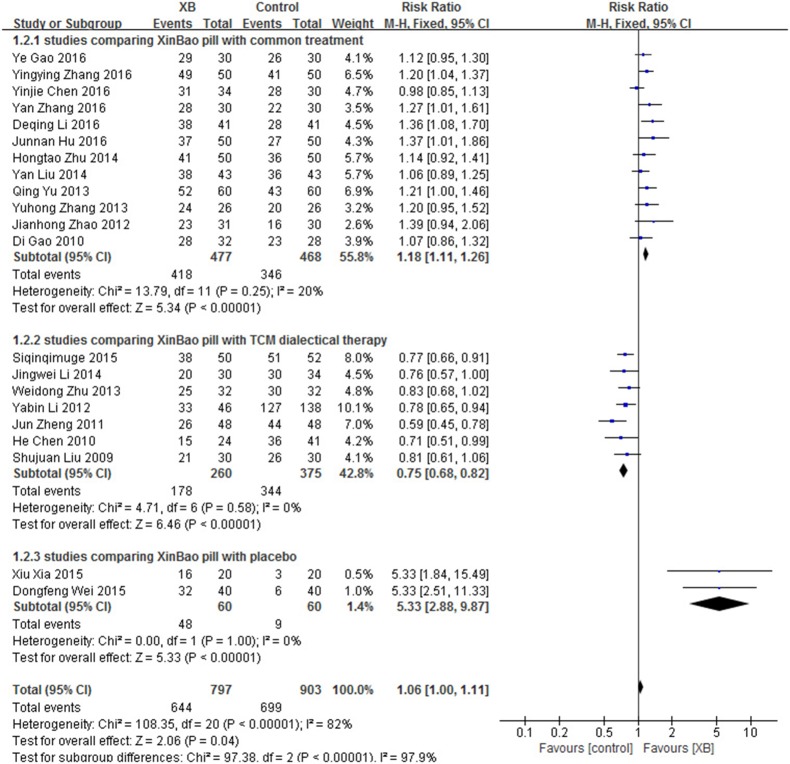
XB for Bradyarrhythmia efficacy by meta-analysis.

Twenty-one studies (Liu et al., 2009, 2014a; Chen et al., 2010, 2016; Di and Zhang, 2010; Zheng, 2011; Li et al., 2012; Zhao, 2012; Yu, 2013; Zhang, 2013, 2016a,b; Zhu, 2013; Li, 2014, 2016; Zhu and Zhang, 2014; Siqingqimuge, 2015; Wei et al., 2015; Xia et al., 2015; Gao et al., 2016; Hu and Zhao, 2016; Zhang and Li, 2016) of XB were included. Courses of the treatment ranged from 1 weeks to 3 months. There were 1,700 patients involved, including 797 in XB group (644 effective cases) and 903 in control group (699 effective cases). Subgroup analysis was performed as substantial heterogeneity was found (*I*^2^ > 50%, *P* < 0.10). It is divided into three subgroup based on the different treatments in control group. There were 12 studies comparing XB with common treatment, 7 studies comparing XB with TCM dialectical therapy, 2 studies comparing XB with placebo. The results showed that differences between the 3 subgroups were obvious (*I*^2^ = 97.9%, *P* < 0.05), indicating the source of heterogeneity. While minimal or no heterogeneity was observed within three subgroups (Figure 5).

As a result, XB can be more effective than common treatment (RR 1.18, 95% CI 1.11-1.26, *P* < 0.00001), as well as placebo (RR 5.33, 95% CI 2.88-9.87, *P* < 0.00001), but less effective than TCM dialectical therapy (RR:0.75, 95% CI 0.68-0.82, *P* < 0.00001) (Figure 5).

### Mahuang-Fuzi-Xixin decoction

MFX, a classical formula from *Treatise on Febrile Diseases (Shang Han Lun in Chinese)*, is comprised of Ephedrae Herba (Ephedra), Aconiti Lateralis Radix Praeparata (Aconitum) and Asari Radix et Rhizoma (Asarum) (Table 1). Mass spectrometric and chromatographic detection identified 52 compounds, including alkaloids, amino acids and organic acids. The main constituents are methyl ephedrine, aconine, songrine, fuziline, neoline, alatisamine, chasmanine, benzoylmesaconine, benzoylaconine, and benzoylhypaconine (Sun et al., 2016). Experiments showed that MFX decoction significantly depressed the expression of IL-6, MCP-1 and TNF-α, and markedly increased expression of IL-10 in serum, indicating the effect of reducing inflammation and increasing antioxidant activities (Tang et al., 2015; Rong et al., 2016). That implied how MFX decoction may affect Bradyarrhythmiaas inflammation is related to myocardial injury which is one of the pathological basis of Bradyarrhythmia (Larsen et al., 2013).

**Figure 6:**
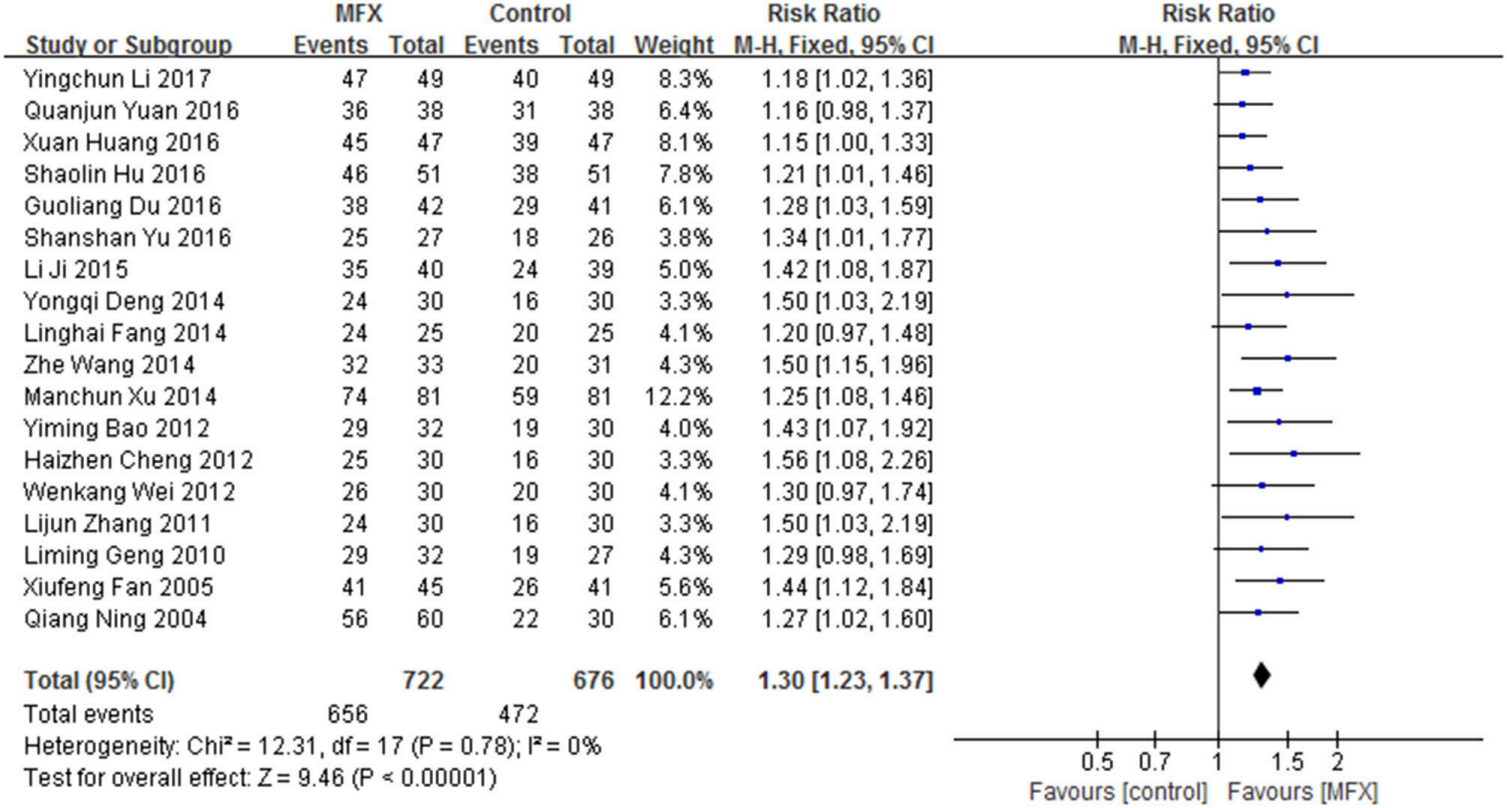
MFX for Bradyarrhythmia efficacy by meta-analysis.

Studies (Ning, 2004; Fan and Yang, 2005; Geng et al., 2010; Zhang L, 2011; Bao, 2012; Cheng, 2012; Wei and Liu, 2012; Deng et al., 2014; Fang et al., 2014; Wang Z, 2014; Xu and Long, 2014; Ji and Zhang, 2015; Du, 2016; Hu and Huang, 2016; Huang and Zhang, 2016; Yu, 2016; Yuan, 2016; Li, 2017) of MFX were included. Courses of the treatment ranged from 2 weeks to 3 months. There were 1398 patients involved, including 722 in MFX group (656 effective cases) and 676 in control group (472 effective cases). Meta-analyse was performed with a fixed-effect model as no significant heterogeneity was found (*I*^2^ < 50%, *P* > 0.10). It showed that MFX was effective in treating Bradyarrhythmia (RR:1.30, 95% CI 1.23 to 1.37, *P* < 0.00001) (Figure 6).

### Zhigancao decoction

ZGC also comes from Treatise on Febrile Diseases (Shang Han Lun in Chinese). It is used as a representative formula to treat almost any kind of arrhythmia containing nine commonly used herbs (Radix glycyrrhizae preparata, Ginger, Cassia Twig, Ginseng, dried rehmannia, Donkey-hide gelatin, Radix Ophiopogonis, Fructus Cannabis, Fructus Ziziphi Jujubae). Recent studies showed that ZGC can effect the Ca^2+^ content in muscle tissue and the excitability of M receptors (Hai et al., 2017). It was reported that ZGC can also treat atrial fibrillation, which is a disease of opposite pathogenesis compared with Bradyarrhythmia. There may exist a two-way regulation effect that requires further study.

**Figure 7:**
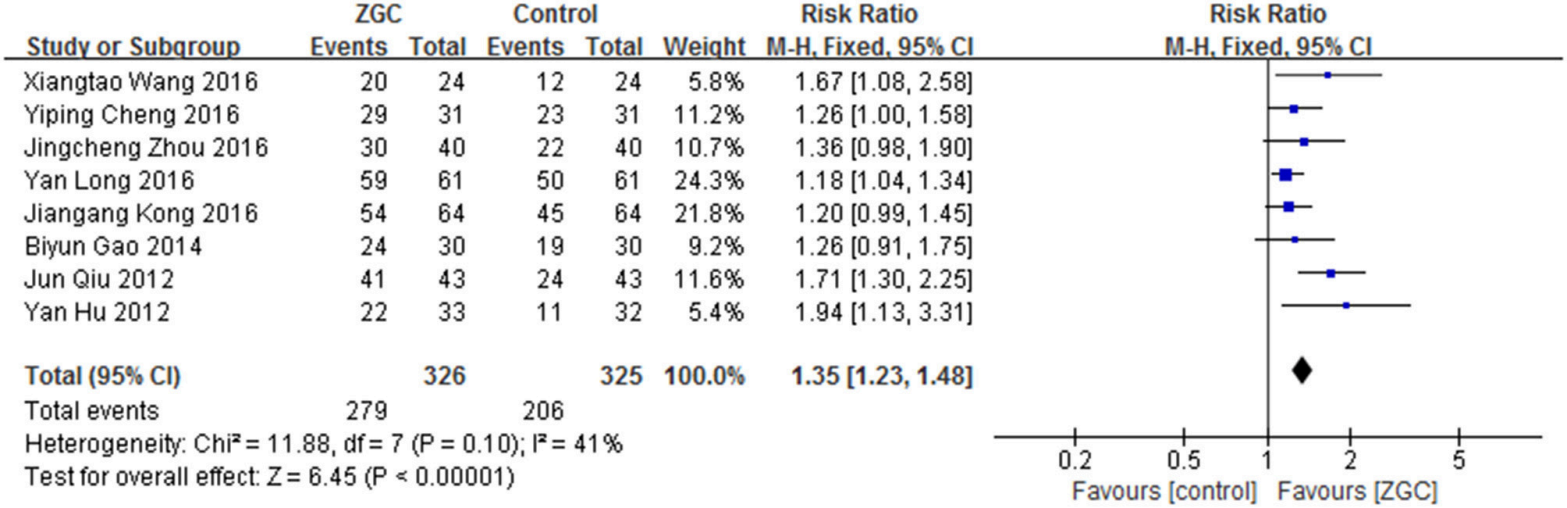
ZGC for Bradyarrhythmia efficacy by meta-analysis.

Eight studies (Hu, 2012; Qiu, 2012; Gao and Chen, 2014; Cheng, 2016; Kong, 2016; Long, 2016; Wang X., 2016; Zhou and Yang, 2016) of ZGC were included. Courses of the treatment ranged from 1 to 3 months. There were 651 patients involved, including 326 in ZGC group (279 effective cases) and 325 in control group (206 effective cases). Meta-analyse was performed with a fixed-effect model as no significant heterogeneity was found (*I*^2^ < 50%, *P* > 0.10). It showed that ZGC was effective in treating Bradyarrhythmia (RR:1.35, 95% CI 1.23 to 1.48, *P* < 0.00001) (Figure 7).

### Shengmai injection

SMI was developed from a classic TCM formula, which is a combination of Panax ginseng, Ophiopogon japonicas and Schisandra chinensis (Table 1). The constituents included ginsenosides, lignans, steroidal saponins and homoisoflavanones (Wu et al., 2011). Proteomics study found that SMI can up-regulate glucose oxidation, TCA cycle and ATP synthesis related proteins, down-regulate proteins catalyzing fatty acid β-oxidation, implying the inhibition of this pathway to avoid high oxygen consumption and modulate the myocardial energy metabolism (Zhan et al., 2015).

**Figure 8:**
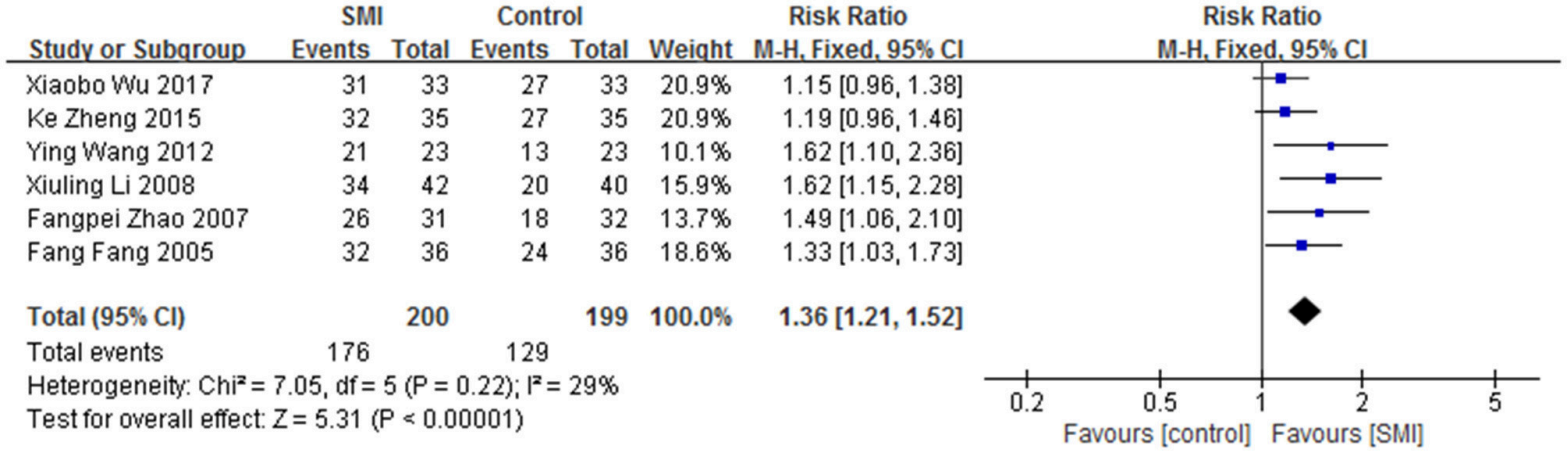
SMI for Bradyarrhythmia efficacy by meta-analysis.

Six studies (Fang, 2005; Zhao and Wang, 2007; Li et al., 2008; Wang et al., 2012; Zheng and Zhao, 2015; Wu, 2017) of SMI were included. Courses of the treatment ranged from 14 to 20 days. There were 399 patients involved, including SMI in SMI group (176 effective cases) and 199 in control group (129 effective cases). Meta-analyse was performed with a fixed-effect model as no significant heterogeneity was found (*I*^2^ < 50%, *P* > 0.10). It showed that SMI was effective in treating Bradyarrhythmia (RR:1.36, 95%CI 1.21 to 1.52, *P* < 0.00001 (Figure 8).

Traditional Chinese medicine has a long history of treating arrhythmia using different kinds of therapies. There are some classic TCM formulas with constant compositions are widely accepted. In this article, we evaluated the effect of six of the most often reported TCM formulas for Bradyarrhythmia with systematic review method, and reviewed their different potential mechanisms as well as therapeutic features (Table 1).

**Figure 9:**
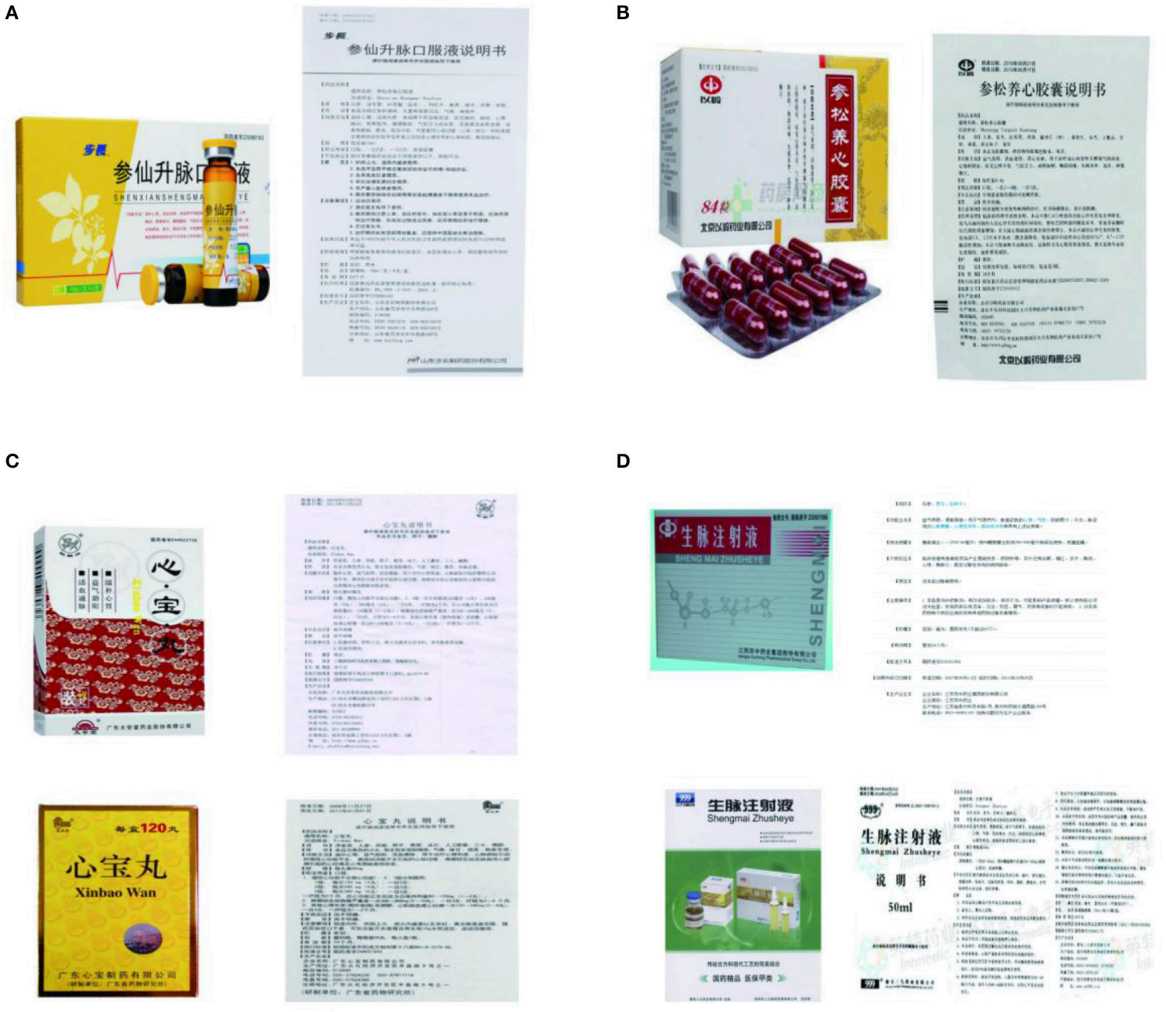
Four included Chinese patent medicine and their drug instructions **(A)** SXSM. **(B)** SSYX. **(C)** XB. **(D)** SMI.

We also assessed the equality of the Chinese patent medicine used in studies. Thirty-two RCTs evaluated the effect of SXSM. As a listed Chinese patent drug with the China's National Registrated No. Z20080183 and detailed drug instruction (containing components, description, dosage, indications etc. Figure 9A), SXSM is manufactured by a single company (Buchang Pharmaceutical Corporation, Heze, China). Thirty-six RCTs evaluated the effect of SSYX, (Shijiazhuang Yiling Pharmaceutical Co., Ltd.) produced the drug. SSYX is a listed Chinese patent drug with the China's National Registrated No. Z20103032 and detailed drug instruction (Figure 9B). XB was evaluated in 21 RCTs, 6 of which didn't specify any information of manufacturer and product batch number (Di and Zhang, 2010; Zhang, 2013; Zhu and Zhang, 2014; Wei et al., 2015; Hu and Zhao, 2016; Zhang and Li, 2016). Ten studies used XB produced by Guangdong Xinbao pharma-tech company (Liu et al., 2009; Chen et al., 2010; Li et al., 2012; Zhu, 2013; Li, 2014, 2016; Siqingqimuge, 2015; Xia et al., 2015; Gao et al., 2016; Zhang, 2016,a,b). Five studies used XB produced by Guangdong Taiantang Pharmaceutical company (Zheng, 2011; Zhao, 2012; Yu, 2013; Liu et al., 2014,a,b; Chen et al., 2016). The ingredients and dosage are basically the same in both XBs (Figure 9C). SMI was evaluated in 6 RCTs. Three studies didn't provide information of manufacturer and product batch number (Wang et al., 2012; Zheng and Zhao, 2015; Wu, 2017). Two studies used SMI produced by Suzhong Yaoye group pharmaceutical limited company (Zhao and Wang, 2007; Li et al., 2008). One study used SMI produced by China resources sanjiu pharmaceutical company (Fang, 2005). The ingredients are basically the same in both SMIs, but the dosages of injection are different (Figure 9D). None of the studies described the details about drug chemical profile or preparation methods.

## Conclusion

In conclusion, TCM formulas showed treatment effect on Bradyarrhythmia while most of the included studies were in low quality. There were a number of clinical trials, but most of them had limitations on small sample size and inadequate descriptions in randomization methods, allocation concealment and blinding methods. Among the six included TCM formulas, SXSM, SSYX, XB, SMI were patent medicine which were authenticated and standardized on marker compounds according to the Chinese Pharmacopeia. But the chemical compounds of the same Chinese patent medicine may not be consistent when produced by different companies and in different batches. XB and SMI were produced by more than one manufacturer, and some of the studies included didn't specify information of manufacturer or product batch number. For herbal compound decoctions like ZGC and MFX, the components among different studies were hard to keep consistent, which made it more important to report full information of the ingredients. Apparently, the importance of components consistency was ignored in most of the current clinical studies. It is highly recommended to give full consideration of it and introduce the source of the species, origin, and concocted methods for each component. Moreover, high performance liquid chromatography, high performance capillary electrophoresis, and gas chromatography should be applied to quantitate the components. It would be helpful for further research and evaluation. On mechanism research, high quality literatures were rare. A lot of efforts had been wasted on repetitious researches of low level which cannot reveal the mechanism of TCM formulas. Therefore, high-quality clinical research and evidence are still needed to evaluate the effectiveness and safety of TCM formulas and innovative researches of mechanism are required as well.

## Author contributions

HS and SL defined the research theme. SL, GT, and JC designed the methods and analyzed the data. JC interpreted the results. SL, YX, and XZ wrote the manuscript, and contributed equally to this work. All authors discussed the results and commented on the manuscript.

### Conflict of interest statement

The authors declare that the research was conducted in the absence of any commercial or financial relationships that could be construed as a potential conflict of interest.

## Abbreviations

HF: heart failure
HR: heart rate
MFX: Mahuang-Fuzi-Xixin decoction
RCT: randomized controlled trial
SMI: Shengmai injection
SB: sinus bradycardia
SSS: sick sinus syndrome
SXSM: Shenxian-shengmai oral liquid
SSYX: Shensong Yangxin capsule
TCM: traditional Chinese medicine
XB: XinBao pill
ZGC: Zhigancao decoction.

## References

[B1] Bai J. Hou P. (2014). Clinical comparative study of shenxian shengmai oral liquid and tongmai yangxin pills in treatment of slow arrhythmia [in Chinese]. J. Pract. Tradit. Chin. Intern. Med. 28, 37–39. 10.13729/j.issn.1671-7813.2014.05.20

[B2] Baman T. S. Kirkpatrick J. N. Romero J. Gakenheimer L. Romero A. Lange D. C.. (2010). Pacemaker reuse: an initiative to alleviate the burden of symptomatic Bradyarrhythmia in impoverished nations around the world. Circulation 122, 1649–1656. 10.1161/CIRCULATIONAHA.110.970483 20956239

[B3] Bao X. Li Q. (2014). Effective observation of shensong yangxin capsule in the therapy of Bradyarrhythmia [in Chinese]. Xian Dai Yang Sheng 20:240.

[B4] Bao Y. (2012). Therapeutic effect of modified mahuang fuzi xixin decoction on patients with Bradyarrhythmia [in Chinese]. Forum Tradit. Chin. Med. 27, 6–7.

[B5] Brignole M. Auricchio A. Baron-Esquivias G. Bordachar P. Boriani G. Breithardt O. A.. (2013). 2013 ESC guidelines on cardiac pacing and cardiac resynchronization therapy: the task force on cardiac pacing and resynchronization therapy of the European Society of Cardiology (ESC). developed in collaboration with the European Heart Rhythm Association (EHRA). Eur. Heart J. 34, 2281–2329. 10.1093/eurheartj/eht150 23801822

[B6] Chen H. Li A. Wu J. (2010). Treatment of Bradyarrhythmia by warming yang and invigorating Qi method: a clinical observation of 41 cases [in Chinese]. J. New Chin. Med. 42, 86–87.

[B7] Chen Y. Zhang Y. Deng M. Ma M. (2016). Thirty-Four cases of sinus Bradycardia after cervical spinal cord injury treated with Xinbao Pills [in Chinese]. Henan Tradit. Chin. Med. 36, 435–436. 10.16367/j.issn.1003-5028.2016.03.0189

[B8] Cheng H. (2012). Clinical analysis of TCM treatment of Bradyarrhythmia [in Chinese]. Gems Health 11, 105–105.

[B9] Cheng Y. (2016). Effective observation of zhigancao decoction in the therapy of Bradyarrhythmia [in Chinese]. Cardiovasc. Dis. J. integr. Trad. Chin. West. Med. 4, 16–17.

[B10] Cho Y. L. Hur S. M. Kim J. Y. Kim J. H. Lee D. K. Choe J.. (2015). Specific activation of insulin-like growth Factor-1 receptor by Ginsenoside Rg5 promotes angiogenesis and vasorelaxation. J. Biol. Chem. 290, 467–477. 10.1074/jbc.M114.603142 25391655 PMC4281749

[B11] Dang S. Huang C. X. Wang X. Wang X. Hu J. Huang H. (2016). Shensong Yangxin (SSYX) ameliorates disordered excitation transmission by suppressing cardiac collagen hyperplasia in rabbits with chronic myocardial infarction. J. Huazhong Univ. Sci. Technol. Med. Sci. 36, 162–167. 10.1007/s11596-016-1560-4 27072956

[B12] Deng Y. Zhang J. Liu G. Sun L. (2014). Effective observation of Mahuang-Fuzi-Xixin decoction in the therapy of Bradyarrhythmia [in Chinese]. J. New Chin. Med. 46, 40–41.

[B13] Di G. Zhang X. (2010). On observation of treatment of Bradyarrhythmia by Xinbao Pill [in Chinese]. China Mod. Doct. 48, 86+137. 10.3969/j.issn.1673-9701.2010.25.053

[B14] Ding Y. (2016). Effective observation of shensong yangxin capsule in the therapy of Bradyarrhythmia [in Chinese]. Cardiovasc. Dis. J. Integr. Tradit. 4, 161+164. 10.3969/j.issn.2095-6681.2016.22.130

[B15] Ding Y. Wei Z. Jin H. (2011). Treating 30 cases of sinus Bradycardia with Shensong Yangxin capsules [in Chinese]. Clin. J. Chillese Med. 3, 53–54. 10.3969/j.issn.1674-7860.2011.24.034

[B16] Dong C. Ma X. (2015). Effective observation of Shenxianshengmai oral liquid in the therapy of Bradyarrhythmia [in Chinese]. Cardiovasc. Dis. J. Integr. Tradit. Chin. West. Med. 3, 53+55.

[B17] Du G. (2016). Treatment of 42 cases of chronic arrhythmia by adding and removing Mahuang Fuzi Xixin Decoction [in Chinese]. Clin. Res. Pract. 1, 106–107.

[B18] Du X. (2014). Clinical experience of Shen Xian Shengmai oral liquid in the treatment of Bradyarrhythmia [in Chinese]. Nei Mongol J. Tradit. Chin. Med. 33:22 10.3969/j.issn.1006-0979.2014.28.024

[B19] Fan X. Yang Y. (2005). 45 cases of sinus Bradycardia treated with Mahuang-fuzi-xixin Decoction [in Chinese]. Shaanxi J. Tradit. Chin. Med. 26, 618–619. 10.3969/j.issn.1000-7369.2005.07.006

[B20] Fang F. (2005). Observation on the therapeutic effect of traditional Chinese medicine combined with intravenous drip in the treatment of Bradyarrhythmia [in Chinese]. Liaoning J. Tradit. Chin. Med. 32, 31–32. 10.3969/j.issn.1000-1719.2005.01.016

[B21] Fang L. Huang H. Sun W. (2014). Clinical observation on the curative effect of modified Mahuang Fuzi Xixin Decoction on treating Bradyarrhythmia [in Chinese]. China Rural Health 15:87.

[B22] Gao B. Chen X. (2014). Modified Zhigancao Decoction in treating arrhythmia of Qi and Yin with deficiency syndrome: a clinical study [in Chinese]. J. Pract. Tradit. Chin. Med. 30, 191–192.

[B23] Gao F. (2017). Treating 70 cases of Bradyarrhythmia with Shensong Yangxin capsules [in Chinese]. Guide China Med. 15, 213–214.

[B24] Gao Y. Tian Y. Wang E. (2005). Simultaneous determination of two active ingredients in Flos daturae by capillary electrophoresis with electrochemiluminescence detection. Anal. Chim. Acta 545, 137–141. 10.1016/j.aca.2005.04.071

[B25] Gao Y. Wang X. Xue M. (2016). Curative effect of Xinbao pill in the treatment of Bradyarrhythmia with chronic heart failure [in Chinese]. Inner Mong. Med. J. 48, 471–472. 10.16096/J.cnki.nmgyxzz.2016.48.04.034

[B26] Gao Z. Wei Y. Wu L. (2015). Observation on the curative effect of Shen Xian Shengmai oral liquid in the treatment of senile Bradyarrhythmia [in Chinese]. Mod. J. Integr. Tradit. Chin. West. Med. 24, 4079–4080. 10.3969/j.issn.1008-8849.2015.36.034

[B27] Ge L. (2010). Shensongyang × in capsule arrhythmia with the clinical effect [in Chinese]. J. Pract. Tradit. Chin. Intern. Med. 24, 52–53. 10.3877/cma.j.issn.2095-6568.2016.01.010

[B28] Ge Y. (2010). Clinical observation of 33 cases of chronic atrial fibrillation with long RR interval treated with Shensong Yangxin Capsule [in Chinese]. China Mod. Med. 17, 53–54.

[B29] Geng L. Li K. Yang Z. (2010). Treatment of 32 cases of chronic arrhythmia due to Coronary Heart Disease by Mahuang-fuzi-xixin decoction [in Chinese]. Henan Traditi. Chin. Med. 30, 853–854.

[B30] Gou Y. (2013). Investigate the clinical efficacy of treatment of fifty sinus Bradycardia by Shensong Yangxin capsules combined with theophylline sustained - release tablets [in Chinese]. Chin. J. Ethnomed. Ethnopharm. 22, 68–69.

[B31] Guo X. (2011). Effect observation of Senxianshengmai oral liquid oral liquid and atropine in treatment of Bradycardia arrhythmias [in Chinese]. J. Med. Forum 32, 128–129.

[B32] Hai Q. S. Yang Y. Q. Meng Z. R. Jing R. U. Jin Y. J. (2017). Effect of Zhigancao Decoction containing serum on rabbit's isolated vascular smooth muscle. Yunnan J. Tradit. Chin. Med. Mater. Med. 38, 21–25.

[B33] Hao P. Jiang F. Cheng J. Ma L. Zhang Y. Zhao Y. (2017). Traditional Chinese medicine for cardiovascular disease: evidence and potential mechanisms. J. Am. Coll. Cardiol. 69, 2952–2966. 10.1016/j.jacc.2017.04.041 28619197

[B34] He L. (2016). Efficacy and security observation of Shenxianshengmai oral liquid in the therapy of sick sinus syndrome [in Chinese]. Yiayao Qianyan 6, 337–338. 10.3969/j.issn.2095-1752.2016.35.299

[B35] Hou P. Chen W. Yu K. Liu N. (2013). Shenxianshengmai oral liquid to cure slow arrhythmia of heart and kidney yang deficiency syndrome randomized controlled study [in Chinese]. J. Pract. Tradit. Chin. Intern. Med, 27, 98–100.

[B36] Hu J. Zhao G. (2016). Clinical observation of adenosine cyclophosphate with Xinbao Pills in treating Bradycardia [in Chinese]. World Clin. Med. 10, 93–95.

[B37] Hu J. Chen S. Hua X. (2012). Clinical research of Shenxian Shengmai oral liquid on sinus Bradycardiac pa_x0002_tients with sick sinus syndrome [in Chinese] Chin. Tradit. Pat. Med. 34, 7–9. 10.3969/j.issn.1001-1528.2012.01.003

[B38] Hu S. Huang L. (2016). Modified Mahuang Fuzi Xixin Decoction in the treatment of Bradyarrhythmia for 51 Cases [in Chinese]. Guangming Journal of Chinese Medicine 31, 2317–2318. 10.3969/j.issn.1003-8914.2016.16.009

[B39] Hu Y. (2012). Clinical observation of Zhigancao Decoction in treatment of Bradyarrhythmia [in Chinese]. J. Sichuan Tradit. Chin. Med. 30, 66–67.

[B40] Huang X. Zhang H. (2016). Clinical observation on the curative effect of modified Mahuang Fuzi Xixin Decoction on treating Bradyarrhythmia [in Chinese]. China Pract. Med. 11, 202–203. 10.14163/j.cnki.11-5547/r.2016.21.142

[B41] Ji L. Zhang Y. (2015). Clinical study on treatment of bradyarrhythmia with modified Mahuang Fuzi Xixin Decoction [in Chinese]. Liaoning J. Tradit. Chin. Med. 42, 986–988. 10.13192/j.issn.1000-1719.2015.05.030

[B42] Jia C. Wang S. (2012). Clinical observation of Shen Song Yangxin Capsule on treatment of Bradycardia and ventricular premature contraction [in Chinese]. J. Clin. Ration. Drug Use 5, 52–53. 10.3969/j.issn.1674-3296.2012.07.041

[B43] Jia J. (2015). Effective observation of Shenxianshengmai oral liquid in the therapy of sick sinus syndrome [in Chinese]. Cardiovasc. Dis. J. Integr. Tradit. Chin. West. Med. 3, 29+31.

[B44] Jiang N. Cen Y. Huang C. Li R. (2012). Clinical observation of Shen Xian Shengmai oral liquid in the treatment of craniocerebral trauma and cerebral cardiac syndrome with sinus Bradycardia [in Chinese]. J. Qiqihar Med. Coll. 33:459 10.3969/j.issn.1002-1256.2012.04.022

[B45] Jin Z. Zhang X. Jin H. Qin X. Li N. Pu J. (2010). Observation of therapeutic effect of Shensong Yangxin Capsule on bradycardiac arrhythmia [in Chinese]. Chin. Tradit. Pat. Med. 32, 1287–1291. 10.3969/j.issn.1001-1528.2010.08.004

[B46] Keaney J. J. Groarke J. D. Galvin Z. McGorrian C. McCann H. A. Sugrue D.. (2013). The Brady Bunch? New evidence for nominative determinism in patients' health: retrospective, population based cohort study. BMJ 347:f6627. 10.1136/bmj.f6627 24336304 PMC3898418

[B47] Kong J. (2016). Zhigancao Decoction treatment of Bradycardia in 64 cases [in Chinese]. Cardiovasc. Dis. J. Integr. Tradit. Chin. West. Med. 4, 51–52. 10.3969/j.issn.2095-6681.2016.23.042

[B48] Larsen B. T. Maleszewski J. J. Edwards W. D. Cooper L. T. Sobonya R. E. Thompson V. E.. (2013). Atrial giant cell myocarditis: a distinctive clinicopathologic entity. Circulation 127, 39–47. 10.1161/CIRCULATIONAHA.112.128900 23183940

[B49] Li B. (2013). Effective observation of Shenxianshengmai oral liquid in the therapy of Bradycardia [in Chinese]. World Health Dig. 10, 234–234.

[B50] Li D. (2016). Effects of trimetazidine combined with heart-pellets on bradyarrhythmia caused by acute inferior myocardial infarction [in Chinese]. Contemporary Med. Symp. 14, 114–115.

[B51] Li G. (2011). Treating 20 cases of Bradycardia with Shengxian Shengmai oral [in Chinese]. Clin. J. Chin. Med. 3, 44–45. 10.3969/j.issn.1674-7860.2011.13.021

[B52] Li G. (2013). Clinical observation on Shensongyangxin Capsules in the treatment of chronic arrhythmia [in Chinese] Med. Front. 11, 94–95. 10.3969/j.issn.2095-1752.2013.11.082

[B53] Li H. (2011). Effect of Shen Xian Shengmai oral liquid combined with theophylline sustained release capsules in the treatment of senile sinus Bradycardia [in Chinese]. People′s Mil. Surg. 54, 124–125.

[B54] Li J. (2014). Treatment of 34 cases of Bradyarrhythmia by warming Yang, invigorating qi and activating blood [in Chinese]. J. Pract. Tradit. Chin. Med. 30:609.

[B55] Li X. Guo S. Du L. (2008). Effect of Shengmai injection in treating elderly patients with Bradyarrhythmia [in Chinese]. Guide China Med. 6, 113–114. 10.3969/j.issn.1671-8194.2008.11.071

[B56] Li Y. (2017). Clinical observation on the curative effect of modified Mahuang Fuzi Xixin Decoction on treating arrhythmiaclinical application of Mahuang Fuzi Xixin Decoction in the treatment of Bradyarrhythmia [in Chinese]. Xin Li Yi Sheng 23, 73–74.

[B57] Li Y. Ren W. Liang J. Dou J. (2012). Fuzheng Tongluo, Quyu Huazhuo treatment of Bradyarrhythmia clinical study [in Chinese]. Jilin J. Tradit. Chin. Med. 32, 810–811. 10.3969/j.issn.1003-5699.2012.08.028

[B58] Li Y. Yin P. Wang L. Liu H. (2016). Observation curative effect on Shensongyangxin capsules in treatment of chronic arrhythmia [in Chinese]. China Continuing Med. Educ. 8, 182–183. 10.3969/j.issn.1674-9308.2016.16.129

[B59] Liang B. (2009). Shensong Yangxin capsule combined with western medicine therapy sinus Bradycardia [in Chinese]. China Prac. Med. 04, 60–61. 10.3969/j.issn.1673-7555.2009.20.037

[B60] Liberati A. Altman D. G. Tetzlaff J. Mulrow C. Gøtzsche P. C. Ioannidis J. P.. (2009). The PRISMA statement for reporting systematic reviews and meta-analyses of studies that evaluate healthcare interventions: explanation and elaboration. BMJ 339:b2700. 10.1136/bmj.b2700 19622552 PMC2714672

[B61] Lin T. (2017). Clinical observation on Shensongyangxin capsules in the treatment of chronic arrhythmia [in Chinese]. China Health Stand. Manag. 8, 88–90. 10.3969/j.issn.1674-9316.2017.09.051

[B62] Liu G. Pan D. Wu Y. Zhuang Y. Yu J. (2016). Efficacy of Shen Xian Shengmai oral liquid in the treatment of bradyarrhythmia in the elderly [in Chinese]. Heilongjiang Med. J. 40, 1145–1146. 10.3969/j.issn.1004-5775.2016.12.035

[B63] Liu H. (2017). Effective observation of Shenxianshengmai oral liquid in the therapy of Bradyarrhythmia [in Chinese]. J. ETCM 26, 530–531. 10.3969/j.issn.1004-745X.2017.03.051

[B64] Liu J. Li Y. (2010). Effective observation of integrative medicine for the treatment of Bradyarrhythmia [in Chinese]. Chin. Commun. Doct. 12:76 10.3969/j.issn.1007-614x.2010.10.091

[B65] Liu J. Chen S. Chen X. (2014). Observation of the effect of permanent pacemaker implantation and Shensong Yangxin capsule in the treatment of Bradyarrhythmia [in Chinese]. Res. Integr. Tradit. Chin. West. Med. 6, 249–250. 10.3969/j.issn.1674-4616.2014.05.008

[B66] Liu M. Zhao S. Wang Y. Liu T. Li S. Wang H. (2015). Identification of multiple constituents in Chinese medicinal prescription Shensong Yangxin capsule by ultra-fast liquid chromatography combined with quadrupole time-of-flight mass spectrometry. J. Chromatogr. Sci. 53, 240–252. 10.1093/chromsci/bmu047 24872525

[B67] Liu S. Yin K. Zhou W. Chen L. (2009). Clinical observation on warming and reinforcing kidney - Yang in treating bradyarrhythmia [in Chinese]. Guangdong Med. J. 30, 1167–1168. 10.3969/j.issn.1001-9448.2009.07.069

[B68] Liu Y. Han W. Liu L. (2014a). Clinical observation on XinBao pill in the treatment of chronic arrhythmia [in Chinese] Guiding J. Tradit. Chin. Med. Pharm. 20, 123–124. 10.13862/j.cnki.cn43-1446/r.2014.04.056

[B69] Liu Y. Li N. Jia Z. Lu F. Pu J. (2014b). Chinese medicine Shensongyangxin is effective for patients with Bradycardia: results of a randomized, double-blind, placebo-controlled multicenter trial. Evid. Based Complement Altern. Med. 2014:605714. 10.1155/2014/605714 24527049 PMC3914313

[B70] Liu Z. Y. Huang J. Liu N. N. Zheng M. Zhao T. Zhao B. C.. (2017). Molecular mechanisms of increased heart rate in Shenxianshengmai-treated Bradycardia rabbits. Chin. Med. J. 130, 179–186. 10.4103/0366-6999.197999 28091410 PMC5282675

[B71] Long Y. (2016). Effective observation of Zhigancao Decoction in the therapy of bradyarrhythmia [in Chinese]. Asia-Pac. Tradit. Med. 12, 132–133. 10.11954/ytctyy.201617063

[B72] Lu X. Liang R. Wang H. (2016). Effective observation of Shenxianshengmai oral liquid in the therapy of sick sinus syndrome [in Chinese]. J. Clin. Psychosom. Dis. 22, 97–97. 10.3969/j.issn.1672-187X.2016.z2.140

[B73] Ma J. Zhu Q. (2010). Clinical effects of Wenyang Buxin capsule treatment on 90 patients with chronic arrhythmia and heart-kidney yang asthenia [in Chinese]. Chin. J. TCM WM Cri.t Care 17, 355–357. 10.3969/j.issn.1008-9691.2010.06.012

[B74] Ma X. Dong C. (2014). Effective observation of Shenxianshengmai oral liquid and Xinbao pill in the therapy of Bradyarrhythmia [in Chinese]. Chin. J. Tradit. Med. Sci. Technol. 21, 36–37.

[B75] Ma Y. (2009). Clinical observation on Shensongyangxin capsules in the treatment of chronic arrhythmia [in Chinese] Qinghai Med. J. 39, 62–63.

[B76] Ma Y. (2011). Efficacy of Shenxian Shengmai oral liquid in the treatment of Bradyarrhythmia in Xining area [in Chinese]. Qinghai Med. J. 41, 22–23.

[B77] Ning Q. (2004). Mahuangfuzixixin Decoction in the treatment of tachycardia: clinical observation of 60 cases of chronic arrhythmia [in Chinese]. Hunan Guiding J. Tradit. Chin. Med. Pharmacol. 10, 29–30. 10.3969/j.issn.1672-951X.2004.06.014

[B78] Pan J. Cui H. (2012). Therapeutic effect of Shensong Yangxin capsule on 600 cases of Bradyarrhythmia [in Chinese]. Chin. Commun. Doctors 14, 185–186. 10.3969/j.issn.1007-614x.2012.26.176

[B79] Qiu J. (2012). Treatment of non-organic sinus Bradycardia with zhigancao decoction combined with atropine: a clinical observation of 43 cases [in Chinese]. Beijing J. Tradit. Chin. Med. 31, 921–922.

[B80] Rong R. Li R. R. Hou Y. B. Li J. Ding J. X. Zhang C. B.. (2016). Mahuang-Xixin-Fuzi decoction reduces the infection of influenza A virus in Kidney-Yang deficiency syndrome mice. J. Ethnopharmacol. 192, 217–224. 10.1016/j.jep.2016.07.017 27401293

[B81] Shen N. Li X. Zhou T. Bilal M. U. Du N. Hu Y.. (2014). Shensong Yangxin capsule prevents diabetic myocardial fibrosis by inhibiting TGF-β1/Smad signaling. J. Ethnopharmacol. 157, 161–170. 10.1016/j.jep.2014.09.035 25267579

[B82] Siqingqimuge (2015). Treatment and nursing of sinus Bradycardia. J. Med. Pharm. Chin. Minor. 21, 80–81. 10.3969/j.issn.1006-6810.2015.06.046

[B83] Sun H. Liu S. (2007). The treatment of 99 cases of coronary heart disease with slow ventricular arrhythmia by Shen Song Yangxin capsule [in Chinese]. Shaanxi J. Tradit. Chin. Med. 28, 1195–1196. 10.3969/j.issn.1000-7369.2007.09.066

[B84] Sun L. Luo Y. (2015). Effective observation of Shenxianshengmai oral liquid in the adjunctive therapy of slow arrhythmias [in Chinese]. Chin. J. Pharmacoepidemiol. 24, 406–408.

[B85] Sun Q. Cao H. Zhou Y. Wang X. Jiang H. Gong L.. (2016). Qualitative and quantitative analysis of the chemical constituents in Mahuang-Fuzi-Xixin decoction based on high performance liquid chromatography combined with time-of-flight mass spectrometry and triple quadrupole mass spectrometers. Biomed. Chromatogr. 30, 1820–1834. 10.1002/bmc.3758 27183898

[B86] Sun X. Cao J. Zhang M. Kong J. Di X. (2012). Shenxian Shengmai oral liquid combined with nitric acid esters in the treatment of chronic arrhythmia in elderly patients with coronary heart disease [in Chinese]. J. Med. Theory Pract. 25, 2362–2363. 10.3969/j.issn.1001-7585.2012.19.017

[B87] Tang H. Li J. Huang Z. (2010). The therapeutic efficacy of Shenxian shengmai oral liquid on patients with sick sinus syndrome [in Chinese]. China Med. 05, 979–980. 10.3760/cma.j.issn.1673-4777.2010.11.005

[B88] Tang F. Tang Q. Tian Y. Fan Q. Huang Y. Tan X. (2015). Network pharmacology-based prediction of the active ingredients and potential targets of Mahuang Fuzi Xixin decoction for application to allergic rhinitis. J. Ethnopharmacol. 176, 402–412. 10.1016/j.jep.2015.10.040 26545458

[B89] Tresch D. D. Fleg L. J. (1986). Unexplained sinus Bradycardia: clinical significance and long-term prognosis in apparently healthy persons older than 40 years. Am. J. Cardiol. 58, 1009–1013. 3490781 10.1016/s0002-9149(86)80029-7

[B90] Wang B. (2016). Clinical observation on Shensongyangxin capsules in the treatment of chronic arrhythmia [in Chinese]. All Health 10:33.

[B91] Wang D. Huang J. Yang H. (2014). Clinical observation on Shensongyangxin capsules in the treatment of chronic arrhythmia [in Chinese] Hebei J. TCM 36, 1374–1375. 10.3969/j.issn.1002-2619.2014.09.055

[B92] Wang W. Wang T. Yuan J. Liu M. Xu Y. Zhang Q. (2014). The effect of Shensongyangxin capsule in patients with paroxysmal atrial fibrillation and Bradycardiac arrhythmia [in Chinese]. Chin. J. Diffic. Compl. Cas. 13, 939–941. 10.3969/j.issn.1671-6450.2014.09.020

[B93] Wang X. (2014). Clinical observation of 30 cases of sinus Bradycardia treated by Shensong Yangxin capsule [in Chinese]. China Prac. Med. 9:186.

[B94] Wang X. (2016). Evaluation on treating arrhythmia with the Zhigancao decoction [in Chinese]. Clin. J. Chin. Med. 8, 35–36. 10.3969/j.issn.1674-7860.2016.32.016

[B95] Wang X. Liu X. (2012). Effect of Shen Xian Shengmai oral liquid combined with small dose of salbutamol in the treatment of elderly sinus Bradycardia [in Chinese]. Chin. J. Clin. Ration. Drug Use 5, 74–75. 10.3969/j.issn.1674-3296.2012.11.057

[B96] Wang Y. Wang J. Yan F. (2012). Clinical research of Shengmai injection on treatment of neonatal Bradycardiac arrhythmia [in Chinese]. Med. Front. 02, 226–227. 10.3969/j.issn.2095-1752.2012.15.241

[B97] Wang Z. (2014). Curative effect of modified Jiahe Fuzi Xixin decoction on treating Bradyarrhythmia [in Chinese]. Chin. J. Convalescent Med. 23:1093 10.13517/j.cnki.ccm.2014.12.017

[B98] Wei D. Zhou H. Xia X. Hu J. (2015). Investigation of the curative effect of Xin Bao pill in the treatment of elderly patients with sinus Bradycardia [in Chinese]. Chin. Commun. Doct. 31, 92+94. 10.3969/j.issn.1007-614x.2015.2.59

[B99] Wei W. Liu H. (2012). 30 cases of Bradyarrhythmia treated with Mahuang-fuzi-xixin Decoction [in Chinese]. Chin. J. Gerontol. 32, 5533–5534. 10.3969/j.issn.1005-9202.2012.24.081

[B100] Wu F. Sun H. Wei W. Han Y. Wang P. Dong T.. (2011). Rapid and global detection and characterization of the constituents in ShengMai San by ultra-performance liquid chromatography–high-definition mass spectrometry. J. Sep. Sci. 34, 3194. 10.1002/jssc.201100253 22012918

[B101] Wu H. (2016). Shensongyangxin capsule treatment efficacy of Bradycardia [in Chinese]. China For. Med. Treat. 30, 128–130. 10.16662/j.cnki.1674-0742.2016.30.128

[B102] Wu H. Wei X. Tian D. (2012). Effective observation of Shenxianshengmai oral liquid in the therapy of Bradyarrhythmia [in Chinese]. Guide China Med. 10:237 10.3969/j.issn.1671-8194.2012.03.181

[B103] Wu J. Zhu J. Yang X. (2015). Observation of the curative effect of s henxian shengmai oral liquid on the elderly with Bradycardiac arrhythmia [in Chinese]. Gansu Med. J. 34, 184–187.

[B104] Wu X. (2017). Effect of Shengmai injection on heart rate and clinical symptoms of patients with Bradyarrhythmia [in Chinese]. Mod. Diagn. Treat. 28, 1195–1196. 10.3969/j.issn.1001-8174.2017.07.012

[B105] Wu Y. Zhang C. Chen Z. (2016). Clinical effect of Shensong Yangxin Capsule on frequent ventricular premature beat and sinus Bradycardia [in Chinese]. Chin. Baby 8:113.

[B106] Xia H. Wang J. Bao C. (2012). Effect of Shensong Yangxin Capsule in the treatment of frequent atrial premature beat with sinus Bradycardia [in Chinese]. Chin. J. Difficult Complic. Cases 11, 123–124. 10.3969/j.issn.1671-6450.2012.02.019

[B107] Xia X. Zhou H. Wei D. (2015). Clinical observation on Xinbao Pill treating 20 cases of senile sinus Bradycardia [in Chinese]. Chin. J. Ethnomed. Ethnopharm. 5, 78–79.

[B108] Xu M. Long H. (2014). Clinical observation on the curative effect of modified Mahuang Fuzi Xixin Decoction on treating Bradyarrhythmia [in Chinese]. Med. Informat. 27:431 10.3969/j.issn.1006-1959.2014.35.675

[B109] Yang H. Ren Q. (2015). Clinical efficacy of shenxian shengmai oral liquid on senile brady arrhythmia [in Chinese]. J. Liaoning Univ. Tradit. Chin. Med. 17, 160–162. 10.13194/j.issn.1673-842x.2015.11.053

[B110] Yang J. H. Han S. J. Ryu J. H. Jang I. S. Kim D. H. (2009). Ginsenoside Rh2 ameliorates scopolamine-induced learning deficit in mice. Biol. Pharm. Bull. 32, 1710–1715. 10.1248/bpb.32.1710 19801832

[B111] Yang Z. Wang F. Guo Q. (2012). Analysis of the effect and adverse reaction of Shen Xian Shengmai oral liquid in the treatment of Bradyarrhythmia [in Chinese]. Shanxi Med. J. 41, 402–403. 10.3969/j.issn.0253-9926.2012.08.056

[B112] Ye J. (2012). Effective observation of Shenxianshengmai oral liquid and theophylline sustained release tablets in the therapy of Sick sinus syndrome [in Chinese]. Chin. J. Integr. Med. Cardio-/Cerebrovasc. Dis. 10, 1176–1177. 10.3969/j.issn.1672-1349.2012.10.015

[B113] Yu Q. (2013). Treating 60 cases of Bradyarrhythmia with XinBao pill [in Chinese]. J. Guiyang Coll. Tradit. Chin. Med. 35, 31–33. 10.3969/j.issn.1002-1108.2013.03.0014

[B114] Yu S. (2016). Effective observation of Mahuang-Fuzi-Xixin decoction in the therapy of Bradyarrhythmia [in Chinese]. China Healthcare Nutr. 26, 341–342.

[B115] Yuan Q. (2016). Clinical observation on the curative effect of modified Mahuang Fuzi Xixin Decoction on treating Bradyarrhythmia [in Chinese]. World Lat. Med. Informat. 16, 208+210. 10.3969/j.issn.1671-3141.2016.100.134

[B116] Zálesák M. Blazícek P. Gablovský I. Ledvényiová V. Barteková M. Ziegelhöffer A.. (2015). Impaired PI3K/Akt signaling as a potential cause of failure to precondition rat hearts under conditions of simulated hyperglycemia. Physiol. Res. 64:633. 25804103 10.33549/physiolres.932883

[B117] Zeng X. Li J. Zeng L. (2010). Treating 118 cases of bradyarrhythmia with Shensong Yangxin capsules [in Chinese]. J. Sichuan Tradit. Chin. Med. 28, 81–82.

[B118] Zhan S. Fan X. Zhang F. Wang Y. Kang L. Li Z. (2015). A proteomic study reveals Shengmai injection preventing cardiac ischemia-reperfusion injury via energy metabolism modulation. Mol. Biosyst. 11, 540–548. 10.1039/C4MB00161C 25427756

[B119] Zhang G. (2016). The curative effect of Shensong Yangxin Capsule in the treatment of sinus Bradycardia with ventricular premature beat [in Chinese]. Cardiovasc. Dis. J. Integr. Tradit. Chin. West. Med. 4, 50–51.

[B120] Zhang L. (2010). Analysis of the curative effect of Shen Song Yangxin capsule in the treatment of Bradyarrhythmia [in Chinese]. Chin. J. Misdiagn. 10:3870.

[B121] Zhang L. (2011). Treatment of 30 Cases of Bradyarrhythmia with Mahuang Fuzi Asarum Decoction and atropine [in Chines]. J. Emerg. Tradit. Chin. Med. 20:468 10.3969/j.issn.1004-745X.2011.03.079

[B122] Zhang M. Li N. Qu X. B. Luo S. Drummen G. P. C. (2016). Total velvet-antler polypeptide extract from Cervus nippon Temminck induces cell proliferation and activation of the PI3K–Akt signalling pathway in human peripheral blood lymphocytes. Anim. Prod. Sci. 56:1008 10.1071/AN15103

[B123] Zhang P. Li D. Wang X. (2010). Analysis of the curative effect of Shen Song Yangxin Capsule in the treatment of sinus Bradycardia and ventricular premature beat [in Chinese]. Chin. J. Misdiagn. 10, 6576–6577.

[B124] Zhang Y. (2013). Clinical analysis of patients with chronic arrhythmia. Guide China Med. 11, 214–215. 10.3969/j.issn.1671-8194.2013.18.154

[B125] Zhang Y. (2014). Clinical observation of Shen Xian Shengmai oral liquid in the treatment of hypothyroid sinus Bradycardia [in Chinese]. Mod. Diagn. Treat. 25, 3732–3733.

[B126] Zhang Y. (2016a). Clinical observation of Xinbao Pill combined with trimetazidine in treatment of chronic heart failure Induced by Coronary Heart Disease [in Chinese]. Hubei J. Tradit. Chin. Med. 38, 3–5.

[B127] Zhang Y. (2016b). Effective observation of Shensong Yangxin Capsule in the therapy of Bradyarrhythmia [in Chinese]. Cardiovasc. Dis. J. integrated Tradit. Chin. West. Med. 4, 30–31.

[B128] Zhang Y. Li S. (2016). Curative effect of Xinbao Pills in treating Coronary Heart Disease with atrial fibrillation and long RR interval [in Chinese]. J. Huaihai Med. 34, 338–339. 10.14126/j.cnki.1008-7044.2016.03.043

[B129] Zhang Y. Song L. Zhao L. Zhang M. Yang C. (2011). Treating 60 cases of Bradyarrhythmia with Shensong Yangxin capsules [in Chinese]. China Med. Herald 8:192 10.3969/j.issn.1673-7210.2011.13.103

[B130] Zhao F. Wang F. (2007). Treatment of sinus Bradycardia with cyclic adenosine monophosphate and Shengmai injection: a clinical analysis of 31 cases [in Chinese]. Chin. J. Coal Ind. Med. 10, 404–405. 10.3969/j.issn.1007-9564.2007.04.033

[B131] Zhao J. (2012). Xinbao pill in the treatment of 31 cases of sick sinus syndrome [in Chinese]. Shaanxi J. Tradit. Chin. Med. 33, 46–47. 10.3969/j.issn.1000-7369.2012.01.024

[B132] Zhao L. (2010). Effect of Shen Song Yangxin Capsule on sinus Bradycardia [in Chinese]. Chin. J. Ethnomed. Ethnopharm. 19:38 10.3969/j.issn.1007-8517.2010.22.032

[B133] Zhao Y. Wei X. Niu J. An C. Liu Y. (2011). Clinical observation of Shensongyangxin capsules in treatment of chronic ar_x0002_rhythmia [in Chinese]. China Med. Herald 8, 71–73. 10.3969/j.issn.1673-7210.2011.03.036

[B134] Zheng J. (2011). Clinical observation of method of tonifying Qi to Warm Yang and promote blood flowon sinus Bradycardia [in Chinese]. Shanxi J. Tradit. Chin. Med. 27, 13–14. 10.3969/j.issn.1000-7156.2011.11.008

[B135] Zheng K. Zhao M. (2015). Shengmai injection in treatment of slow arrhythmia [in Chinese]. J. Changchun Univ. Chin. Med. 31, 118–120. 10.13463/j.cnki.cczyy.2015.01.040

[B136] Zhou J. (2015). Clinical observation of using Shenxian Shengmai Oral liquid to treat 34cases of chronic arrhythmia [in Chinese]. J. Sichuan Tradit. Chin. Med. 33, 115–117.

[B137] Zhou J. Yang G. (2016). Clinical efficacy analysis of Fried Glycyrrhizae decoction in treating bradyarrhythmias [in Chinese]. Clin. J. Chin. Med. 8, 38–39. 10.3969/j.issn.1674-7860.2016.09.019

[B138] Zhou X. Ma B. Wu J. (2016). ShenXian ShengMai Oral liquid jointed with Aminophy7Iline in treating 32 patients with Bradycardiac Arrhythmia [in Chinese]. West. J. Tradit. Chin. Med. 29, 84–85. 10.3969/j.issn.1004-6852.2016.06.032

[B139] Zhu H. (2016). Clinical observation of Mexiletinc combined with Shensongyangxin capsule in the treatment of Bradyarrhythmia [in Chinese]. Gansu Sci. Technol. 32, 128–129+123. 10.3969/j.issn.1000-0952.2016.04.046

[B140] Zhu H. Zhang C. (2014). The clinical observation of xinbao pill in the treatment of 50 cases of slow arrhythmia [in Chinese]. Chin. Med. Innovat. 11, 104–106. 10.3969/j.issn.1674-4985.2014.35.038

[B141] Zhu W. (2013). A comparative study on curative effect of Xinfukang Capsule and Xinbao Pills in treating Bradyarrhythmia [in Chinese]. Pract. J. Cardiac Cereb. Pneumal Vascul. Dis. 21, 44–45. 10.3969/j.issn.1008-5971.2013.10.019

[B142] Zhu Z. (2011). Observation of the efficacy of Shen Song Yangxin Capsule in the therapeutic room with premature beat and sinus Bradycardia [in Chinese]. Chin. J. Integr. Med. Cardio/Cerebrovasc. Dis. 9, 490–491. 10.3969/j.issn.1672-1349.2011.04.055

[B143] Zhuo J. (2013). Curative observation of using Shenxian Shengmai oral liquid to treat 54 patients with Bradycardia [in Chinese]. J. Sichuan Tradit. Chin. Med. 31, 88–89.

