# Supplementary material for: Traditional Chinese Medicine for Bradyarrhythmia: Evidence and Potential Mechanisms
Source: Front Pharmacol. 2018 Apr 9;9:324. doi: 10.3389/fphar.2018.00324 (PMC5900793; doi:10.3389/fphar.2018.00324)
Supplement: Supplementary file 2 [file DataSheet1.DOCX]

Components of the included TCM formulas

| **Name of formula** | **Chinese name** | **Accepted name** | **Family** | **Taxonomic source** | **Web links** |
| --- | --- | --- | --- | --- | --- |
| Shenxian-shengmai oral liquid  (SXSM) | Ren shen | Panax ginseng C.A.Mey. | Araliaceae | World Checklist | http://mpns.kew.org/mpns-portal/plantDetail?plantId=146697&query=Panax+ginseng+C.A.Mey.&filter=&fuzzy=false&nameType=all&dbs=wcs |
|  | Yin yanghuo | Epimedium brevicornu Maxim. | Berberidaceae | World Checklist: unpublished records | http://mpns.kew.org/mpns-portal/plantDetail?plantId=791222&query=Epimedium+brevicornu+Maxim.&filter=&fuzzy=false&nameType=latin&dbs=wcsCmp |
|  | Bu guzhi | Cullen corylifolium (L.) Medik. | Fabaceae | World Checklist: unpublished records | http://mpns.kew.org/mpns-portal/plantDetail?plantId=747305&query=Psoralea+corylifolia+Linn.&filter=&fuzzy=false&nameType=latin&dbs=wcsCmp |
|  | Gou qi | Lycium barbarum L. | Solanaceae | World Checklist: unpublished records | http://mpns.kew.org/mpns-portal/plantDetail?plantId=499019&query=Lycium+barbarum+L.&filter=&fuzzy=false&nameType=latin&dbs=wcsCmp |
|  | Ma huang | Ephedra sinica Stapf | Ephedraceae | World Checklist | http://mpns.kew.org/mpns-portal/plantDetail?plantId=333041&query=Ephedra+sinica+Stapf&filter=&fuzzy=false&nameType=latin&dbs=wcs |
|  | Xi xin | Asarum sieboldii Miq. | Aristolochiaceae | World Checklist: unpublished records | http://mpns.kew.org/mpns-portal/plantDetail?plantId=654443&query=Asarum+sieboldii+Miq.&filter=&fuzzy=false&nameType=latin&dbs=wcsCmp |
|  | Dan shen | Salvia miltiorrhiza Bunge | Lamiaceae | World Checklist | http://mpns.kew.org/mpns-portal/plantDetail?plantId=183206&query=Salvia+miltiorrhiza+Bunge&filter=&fuzzy=false&nameType=latin&dbs=wcs |
|  | Shui zhi | Whitmania Blanchard, 1888 | Hirudinidae | [The National Checklist of Taiwan](https://www.gbif.org/dataset/1ec61203-14fa-4fbd-8ee5-a4a80257b45a) | https://www.gbif.org/species/4810287 |
| Shensong Yangxin Capsule  (SSYX) | Ren shen | Panax ginseng C.A.Mey. | Araliaceae | World Checklist | http://mpns.kew.org/mpns-portal/plantDetail?plantId=146697&query=Panax+ginseng+C.A.Mey.&filter=&fuzzy=false&nameType=all&dbs=wcs |
|  | Mai dong | Ophiopogon japonicus (Thunb.) Ker Gawl. | Asparagaceae | World Checklist | http://mpns.kew.org/mpns-portal/plantDetail?plantId=279475&query=Ophiopogon+japonicus+&filter=&fuzzy=false&nameType=all&dbs=wcs |
|  | Shan zhuyu | Cornus officinalis Siebold & Zucc. | Cornaceae | World Checklist | http://mpns.kew.org/mpns-portal/plantDetail?plantId=47459&query=Cornus+officinalis+Sieb.+et+Zucc.&filter=&fuzzy=false&nameType=all&dbs=wcs |
|  | Dan shen | Salvia miltiorrhiza Bunge | Lamiaceae | World Checklist | http://mpns.kew.org/mpns-portal/plantDetail?plantId=183206&query=Salvia+miltiorrhiza+Bunge&filter=&fuzzy=false&nameType=all&dbs=wcs |
|  | Suan zaoren | Ziziphus jujuba Mill. | Rhamnaceae | World Checklist: unpublished records | http://mpns.kew.org/mpns-portal/plantDetail?plantId=470699&query=ziziphus+jujuba+&filter=&fuzzy=false&nameType=all&dbs=wcsCmp |
|  | Sang jisheng | Taxillus sutchuenensis (Lecomte) Danser | Loranthaceae | World Checklist: unpublished records | http://mpns.kew.org/mpns-portal/plantDetail?plantId=441678&query=Taxillus+sutchuenensis+%28Lecomte%29+Danser&filter=&fuzzy=false&nameType=all&dbs=wcsCmp |
|  | Chi shao | Paeonia anomala subsp. veitchii (Lynch) D.Y.Hong & K.Y.Pan | Paeoniaceae | World Checklist | http://mpns.kew.org/mpns-portal/plantDetail?plantId=518977&query=Paeonia+veitchii+Lynch&filter=&fuzzy=false&nameType=all&dbs=wcs |
|  | Tu biechong | Eupolyphaga sinensis (Walker, 1868) | Corydiidae Saussure, 1864 | Walker, F. 1868. Catalogue of the Specimens of Blattariae in the Collection of the British Museum, British Museum (Natural History), London 239  in [Cockroach Species File](https://www.gbif.org/dataset/3e812f13-bd5f-46b6-9bae-710766be526d) | https://www.gbif.org/species/100473595 |
|  | Gan song | Nardostachys jatamansi (D.Don) DC. | Caprifoliaceae | World Checklist: unpublished records | http://mpns.kew.org/mpns-portal/plantDetail?plantId=382349&query=Nardostachys+DC.&filter=&fuzzy=false&nameType=all&dbs=wcsCmp |
|  | Huang lian | Coptis chinensis Franch. | Ranunculaceae | World Checklist: unpublished records | http://mpns.kew.org/mpns-portal/plantDetail?plantId=736105&query=Coptis+chinensis+Franch.&filter=&fuzzy=false&nameType=all&dbs=wcsCmp |
|  | Wu weizi | Kadsura longipedunculata Finet & Gagnep.; | Schisandraceae | World Checklist | http://mpns.kew.org/mpns-portal/plantDetail?plantId=377810&query=Kadsura+longipedunculata+&filter=&fuzzy=false&nameType=all&dbs=wcs |
|  | Long gu | OsDraconis | Drgonsbones |  | https://baike.baidu.com/item/%E9%BE%99%E9%AA%A8/12644668 |
| XinBao pill  (XB) | Yang jinhua | Datura metel L. | Solanaceae | WCSP (in review) | http://www.theplantlist.org/tpl1.1/record/kew-2757816 |
|  | Lu rong | Cornu cervi pantotrichum(Huang et al., 2017) | cornu cervi pantotrichum |  | https://baike.so.com/doc/1713898-1811958.html |
|  | Ren shen | Panax ginseng C.A.Mey. | Araliaceae | World Checklist | http://mpns.kew.org/mpns-portal/plantDetail?plantId=146697&query=Panax+ginseng+C.A.Mey.&filter=&fuzzy=false&nameType=all&dbs=wcs |
|  | Fu zi | Aconitum carmichaeli Debeaux | Ranunculaceae | World Checklist: unpublished records | http://mpns.kew.org/mpns-portal/plantDetail?plantId=618501&query=Aconitum+carmichaeli+Debx.&filter=&fuzzy=false&nameType=all&dbs=wcsCmp |
|  | Rou gui | Cinnamomum cassia (L.) J.Presl | Lauraceae | World Checklist: unpublished records | http://mpns.kew.org/mpns-portal/plantDetail?plantId=721201&query=Cinnamomum+cassia+Presl&filter=&fuzzy=false&nameType=all&dbs=wcsCmp |
|  | San qi | Panax notoginseng (Burkill) F.H.Chen | Araliaceae | World Checklist | http://mpns.kew.org/mpns-portal/plantDetail?plantId=146751&query=Panax+notoginseng%C2%A0%28Burk.%29F.H.Chen&filter=&fuzzy=false&nameType=all&dbs=wcs |
|  | She xiang | Abelmoschus moschatus Medik. | Malvaceae | World Checklist: unpublished records | http://mpns.kew.org/mpns-portal/plantDetail?plantId=609599&query=Moschus&filter=&fuzzy=false&nameType=all&dbs=wcsCmp |
|  | Chan su | Venenum Bufonis(Bi et al., 2016) | cinobufagin venom toad |  | https://baike.baidu.com/item/%E8%9F%BE%E9%85%A5#1_8 |
|  | Bing pian | Dryobalanops aromatica  C.F.Gaertn. | Dipterocarpaceae | [World Checklist: unpublished records](http://mpns.kew.org/mpns-portal/reference?reference=World%20Checklist:%20unpublished%20records&query=Dryobalanops+aromatica+Gaertn.+f.+&filter=&fuzzy=false&nameType=all) | http://mpns.kew.org/mpns-portal/plantDetail?plantId=778746&query=Dryobalanops+aromatica+Gaertn.+f.+&filter=&fuzzy=false&nameType=all&dbs=wcsCmp |
| Mahuang-Fuzi-Xixin decoction  （MFX） | Ma huang | Ephedra sinica Stapf | Ephedraceae | World Checklist | http://mpns.kew.org/mpns-portal/plantDetail?plantId=333041&query=Ephedra+sinica+Stapf&filter=&fuzzy=false&nameType=all&dbs=wcs |
|  | Fu zi | Aconitum carmichaeli Debeaux | Ranunculaceae | World Checklist: unpublished records | http://mpns.kew.org/mpns-portal/plantDetail?plantId=618501&query=Aconitum+carmichaeli+Debx.&filter=&fuzzy=false&nameType=all&dbs=wcsCmp |
|  | Xi xin | Asarum sieboldii Miq. | Aristolochiaceae | World Checklist: unpublished records | http://mpns.kew.org/mpns-portal/plantDetail?plantId=654443&query=Asarum+sieboldii+Miq.&filter=&fuzzy=false&nameType=latin&dbs=wcsCmp |
| Zhigancao decoction  (ZGC) | Gan cao | Glycyrrhiza uralensis Fisch. ex DC. | Fabaceae | World Checklist: unpublished records | http://mpns.kew.org/mpns-portal/plantDetail?plantId=827776&query=Glycyrrhiza+uralensis+Fisch.&filter=&fuzzy=false&nameType=all&dbs=wcsCmp |
|  | Sheng jiang | Zingiber officinale Roscoe | Zingiberaceae | World Checklist | http://mpns.kew.org/mpns-portal/plantDetail?plantId=273361&query=Zingiber+officinale+Roscoe&filter=&fuzzy=false&nameType=all&dbs=wcs |
|  | Gui zhi | Cinnamomum cassia (L.) J.Presl | Lauraceae | World Checklist: unpublished records | http://mpns.kew.org/mpns-portal/plantDetail?plantId=721201&query=Cinnamomum+cassia+Presl&filter=&fuzzy=false&nameType=all&dbs=wcsCmp |
|  | Ren shen | Panax ginseng C.A.Mey. | Araliaceae | World Checklist | http://mpns.kew.org/mpns-portal/plantDetail?plantId=146697&query=Panax+ginseng+C.A.Mey.&filter=&fuzzy=false&nameType=all&dbs=wcs |
|  | Di huang | Rehmannia glutinosa (Gaertn.) DC. | Orobanchaceae | World Checklist: unpublished records | http://mpns.kew.org/mpns-portal/plantDetail?plantId=527243&query=Rehmannia+glutinosa+&filter=&fuzzy=false&nameType=all&dbs=wcsCmp |
|  | E jiao | Colla corii asini (Donkey-hide gelatin, E-Jiao)(Wu et al., 2016) | Ejiao |  | https://baike.baidu.com/item/%E9%98%BF%E8%83%B6 |
|  | Mai dong | Ophiopogon japonicus (Thunb.) Ker Gawl. | Asparagaceae | World Checklist | http://mpns.kew.org/mpns-portal/plantDetail?plantId=279475&query=Ophiopogon+japonicus+&filter=&fuzzy=false&nameType=all&dbs=wcs |
|  | Ma ren | Cannabis sativa L. | Cannabaceae | World Checklist: unpublished records | http://mpns.kew.org/mpns-portal/plantDetail?plantId=696480&query=Cannabis+sativa+L.&filter=&fuzzy=false&nameType=all&dbs=wcsCmp |
|  | Da zao | Ziziphus jujuba Mill. | Rhamnaceae | World Checklist: unpublished records | http://mpns.kew.org/mpns-portal/plantDetail?plantId=470699&query=Ziziphus+zizyphus.&filter=&fuzzy=false&nameType=all&dbs=wcsCmp |
| Shengmai injection  (SMI) | Ren shen | Panax ginseng C.A.Mey. | Araliaceae | World Checklist | http://mpns.kew.org/mpns-portal/plantDetail?plantId=146697&query=Panax+ginseng+C.A.Mey.&filter=&fuzzy=false&nameType=all&dbs=wcs |
|  | Mai dong | Ophiopogon japonicus (Thunb.) Ker Gawl. | Asparagaceae | World Checklist | http://mpns.kew.org/mpns-portal/plantDetail?plantId=279475&query=Ophiopogon+japonicus+&filter=&fuzzy=false&nameType=all&dbs=wcs |
|  | Wu weizi | Schisandra chinensis (Turcz.) Baill. | Schisandraceae | World Checklist | http://mpns.kew.org/mpns-portal/plantDetail?plantId=381262&query=Schisandra+chinensis&filter=&fuzzy=false&nameType=all&dbs=wcs |

Notes: some of the components of traditional Chinese medicine formulas come from animals or mineral, which do not have specific Taxonomic source.


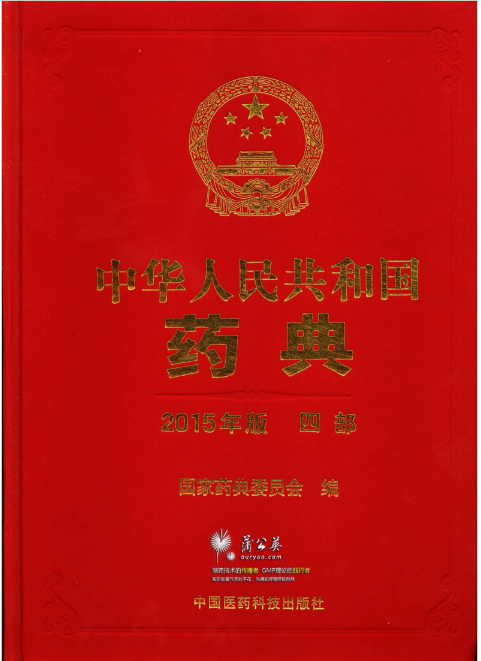

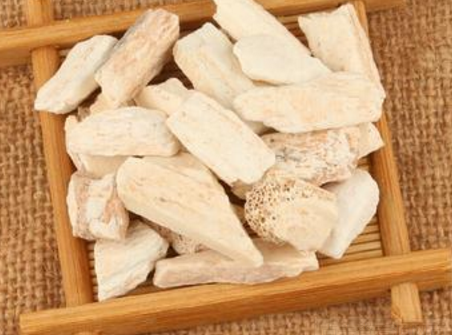


Os Draconis

Os Draconis is a fine powder from the calcined skeleton fossil of a Mastodon containing anxiolytic, a sedative in Oriental medicine. The calcined skeletons, found mostly in Shanxi, Gansu, Heibei and Inner Mongolia, require special preparations to remove impurities before the powder can be used. Because of its sedative prosperities, Os Draconis helps the nervous system, and when mixed with Fructus Ziziphi Jujubae, Glycyrrhizae Radix, and Ostrea Gigas, the powder can calm restlessness, seminal leakage, an overactive bladder and severe incontinence. Several TCM doctors prescribe Os Draconis to help with performance anxiety disorders and neuropsychopathic dysfunctions(Pharmacopoeia, 2015）.


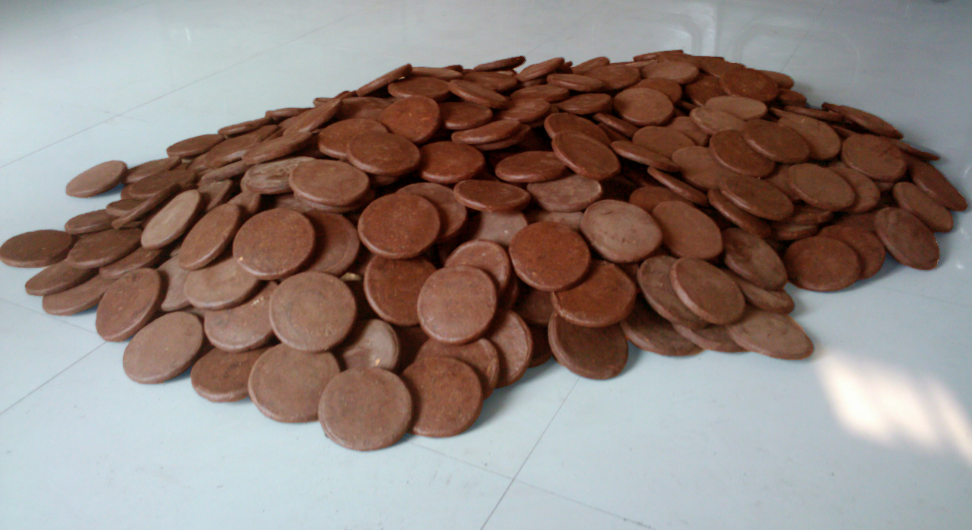


Venenum Bufonis

Venenum Bufonis (VB), also called toad venom or Chan Su in China, Senso in Japan and Somso in korea, derives from the dried white secretion of the auricular and skin glands of Bufo bufo gargarizans Cantor or Bufo melanostictus Schneider (Pharmacopoeia, 2010). The crude drug form of VB, coming from Bufo bufo gargarizans Cantor, was purchased from Bozhou Medicine Company (Anhui, China) and authenticated by professor De-an Guo (Shanghai Institute of Materia Medica, Chinese Academy of sciences). The voucher specimens were deposited at Shanghai Research Center for Modernization of Traditional Chinese Medicine. Desacetylbufotalin, arenobufagin, hellebrigenin, telocinobufagin, bufotalin, cinobufotalin, bufalin, resibufogenin, cinobufagin were isolated from VB by the authors. Their structures were fully identified by UV, MS and NMR with purities of more than 95%.(Bi et al., 2016)


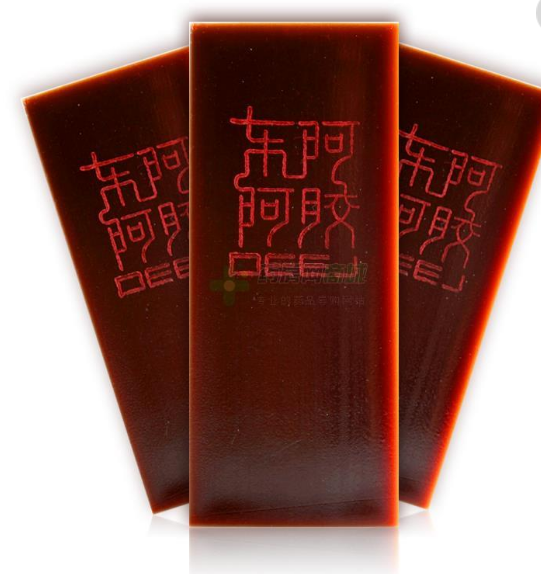

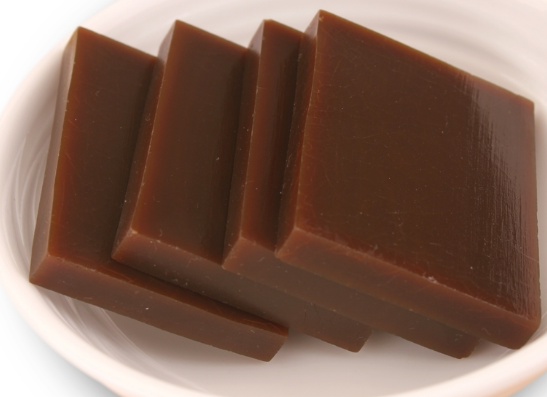


Colla corii asini

Colla corii asini (Donkey-hide gelatin, E-Jiao) is one of the wellknown traditional Chinese medicines (TCMs) and nutritional supplements for over two thousand years (Zhonghua Renmin Gongheguo wei sheng bu yao dian wei yuan hui, 1997). It is a solid glue prepared from the skin of Equus asinus by decoction and concentration, and mainly contains amino acids, microelements, and small molecular weight collagen hydrolysate (Zhonghua Renmin Gongheguo wei sheng bu yao dian wei yuan hui, 1997). A recent review reported that about 58 compounds or chemical constituents were isolated from colla corii asini over the past few decades, including amino acids, proteins/gelatins, polysaccharides, volatile substances, and inorganic substances . Some pharmacological properties of colla corri asini include sedation, anticoagulation, vasodilatation, hematopoiesis, as well as enhancement of cellular immunity and radio-protection. Consequently, it has been widely used to treat gynecologic diseases (i.e., dysmenorrhea, menoxienia, metrorrhagia, abortion) and chronic diseases (i.e., anxiety, insomnia, apostaxis, hemoptysis, hematuria, hemafecia) (Bi et al., 2016).


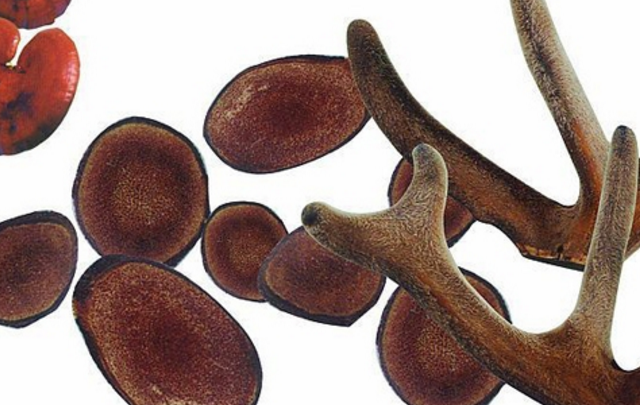


Cornu cervi pantotrichum

Cornu cervi pantotrichum (CCP), also known as Lu Rong, is harvested from deer antlers. The Compendium of Materia Medica (Bencao Gangmu) written by Li Shi-Zhen during the Ming Dynasty (430 years ago) recorded that CCP had several medical benefits, such as promotion of marrow hematopoiesis, anti-inflammation, kidney nourishment, and amelioration of wasting and vertigo. The major nutrient components or constituents of CCP include several minerals (Ca, P, K, Al, Zn, Cu and Fe), amino acids, carbohydrates, phospholipids, polypeptides, proteins and cell growth factors. In laboratory-based experimental research, the CCP has been demonstrated to have a broad-spectrum of bioactivities including immune modulation, anti-fatigue, anti-osteoporosis, anti-inflammation, hematopoietic modulation, chronic wound healing and the promotion of hair growth(Huang et al., 2017) .This herb is the hairy, non-ossificying young born of male deer or stag of Cervus nippon Temminck or C. elaphus Linnaeus, vertebrae of the family Cervidae. Commonly, the former is called Hua Lu Rong (pilose antler from Cervus nippon Temminck), and the latter is Ma Lu Rong (that from C. elaphus Linnaeus).

**References:**

Bi, Q.R., Hou, J.J., Qi, P., Ma, C.H., Shen, Y., Feng, R.H., Yan, B.P., Wang, J.W., Shi, X.J., Zheng, Y.Y., Wu, W.Y., and Guo, D. (2016). Venenum Bufonis induces rat neuroinflammation by activiating NF-kappaB pathway and attenuation of BDNF. *J. Ethnopharmacol.* 186, 103-110. doi: 10.1016/j.jep.2016.03.049.

Huang, W.C., Huang, C.C., Chuang, H.L., Chiu, C.C., Chen, W.C., and Hsu, M.C. (2017). Cornu cervi pantotrichum supplementation improves physiological adaptions during intensive endurance training. *J. Vet. Med. Sci.* 79, 674-682. doi: 10.1292/jvms.16-0623.

Wu, H., Ren, C., Yang, F., Qin, Y., Zhang, Y., and Liu, J. (2016). Extraction and identification of collagen-derived peptides with hematopoietic activity from Colla Corii Asini. *J. Ethnopharmacol.* 182, 129-36. doi: 10.1016/j.jep.2016.02.019.
